# Protein–Protein Interactions Modulate a Key Branch Point in Monoterpene Indole Alkaloid Biosynthesis

**DOI:** 10.1021/acschembio.5c00485

**Published:** 2026-01-04

**Authors:** Samuel C. Carr, Allwin McDonald, Chloe Langley, Veit Grabe, Klaus Gase, Sarah E. O’Connor

**Affiliations:** † Department of Natural Product Biosynthesis, 28298 Max Planck Institute for Chemical Ecology, 07745 Jena, Germany; § Microscopic Imaging Service Group, Max Planck Institute for Chemical Ecology, 07745 Jena, Germany

## Abstract

Biosynthetic pathways of specialized metabolites utilize protein–protein interactions (PPIs) to facilitate metabolic flux and sequester reactive intermediates. The monoterpene indole alkaloid pathway of *Catharanthus roseus* contains several metabolic branch points that may be mediated via transient PPIs. We investigated one branch point of this pathway that is responsible for the conversion of the intermediate dehydrosecodine into three possible cyclized alkaloid scaffolds, which act as intermediates en route to medicinally important alkaloids, such as vinblastine. We verified previously observed PPIs between reductase-cyclase pairs and additionally uncovered PPIs between evolutionarily related protein homologues. Through structural analysis of dehydrosecodine cyclases, we identified surface residues that appear to mediate interaction with the upstream reductase. We then demonstrated, via *in vitro* competition assays, that these residues impact the distribution of downstream products. These results highlight the significance of transient PPIs in the control and regulation of specialized metabolite pathways.

## Graphical abstract

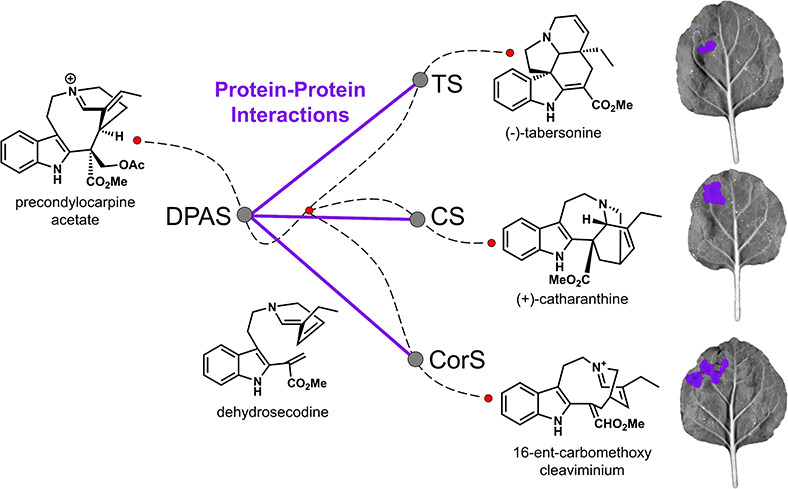

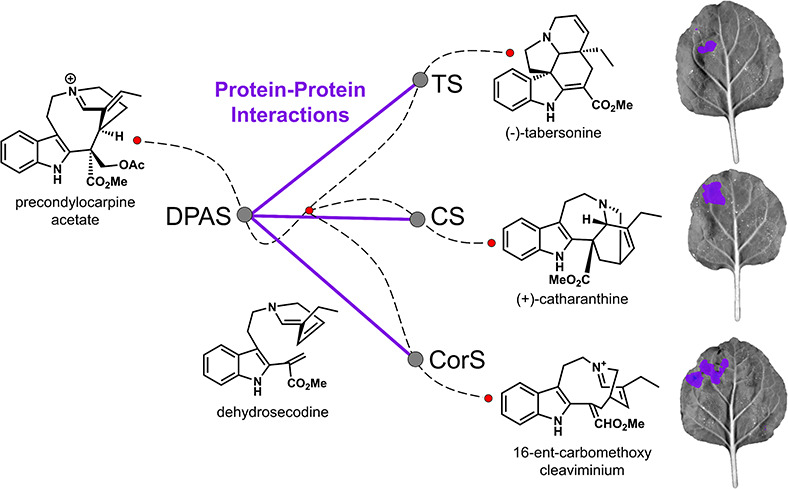

Protein–protein interactions (PPIs) are crucial elements in biosynthetic pathways. Alongside gene clustering, gene fusion, enzyme colocalization, and metabolite sequestration, PPIs represent one way to direct metabolic flux^1,2^ and limit promiscuous side reactions of intermediates.^3–5^ In plants, PPIs have been shown to enhance the efficiency and fidelity of flavonoid,^5–7^ isoflavonoid,^8^ polyketide,^9^ camalexin,^10^ sporopollenin,^11^ glucosinolate,^4^ tricarboxylic acid,^12^ cyanogenic glucoside,^13^ and bitter acid^14^ biosynthesis (several informative reviews^1,2,15^ ). Some of these PPIs facilitate channeling of metabolites from one pathway enzyme active site to the next (metabolons).^16^ Metabolic branch points represent key decision-making steps within cells, and PPIs are known to play a role in the decision process. Transient interactions between enzymes may facilitate dynamic assembly/disassembly to adapt to the ever-changing environment of living cells. Mediation of plant biosynthetic branch points through PPIs has been reported in flavonoid^6,17^ and benzoxazinoid^18^ biosynthesis. In these systems, dynamic association and/or dissociation of branch point enzymes modulates the pathway flux. Such transient PPIs have been detected using a variety of methods, including protein NMR,^9^ chemical cross-linking,^9^ yeast two-hybrid,^5,11,12,14^ biomolecular fluorescence complementation (BiFC),^5^ split-luciferase,^12^ or Förster resonance energy transfer (FRET).^10,11,13^

Monoterpene indole alkaloids (MIAs) are a large and diverse class of plant specialized metabolites found in several plant families, including the *Apocynaceae*.^19^ MIAs exhibit a range of desirable properties, including anticancer, antimalarial, opioid-receptor-binding, and psychedelic bioactivities.^20–23^ The tumor suppressants vinblastine and vincristine, which are dimeric MIAs isolated from *Catharanthus roseus*, are formed by the coupling of the monomers vindoline and catharanthine, whose biosynthetic pathways diverge at a key branch point marked by the production of the reactive biosynthetic intermediate dehydrosecodine (Figure 1). A medium-chain dehydrogenase/reductase named dihydroprecondylocarpine acetate synthase (DPAS) catalyzes the reduction of precondylocarpine acetate (PCA) to dihydro-PCA, which then spontaneously deacetoxylates to form dehydrosecodine (Figure 1).^24^ Dehydrosecodine can rearrange to form the compound angryline, be further reduced by DPAS,^25^ or be intercepted by cyclases tabersonine synthase (TS) and catharanthine synthase (CS). TS and CS catalyze the formation of the aspidosperma-type MIA tabersonine (precursor to vindoline) and the iboga-type MIA catharanthine, respectively, in *C. roseus*.^24^ A third cyclase, coronaridine synthase (CorS), catalyzes the formation of the iboga-type MIA 16-ent-carbomethoxycleaviminium (16-cmc) in *C. roseus* and members of the *Tabernaemontaneae* lineage such as *Tabernanthe iboga*.^26^ Further reduction of 16-cmc by DPAS results in formation of the iboga-type MIA (−)-coronaridine, a precursor to the medicinally important antiaddiction agent ibogaine. Recent work suggests that TS, CS, and CorS evolved from a single ancestral α/β-hydrolase.^27^ Homologues of DPAS, TS (also named TabS), and CorS from both *C. roseus* and *T. iboga* have been well-characterized, including *Cr*DPAS, *Ti*DPAS1, *Ti*DPAS2; *Cr*TS and *Ti*TabS; *Cr*CorS and *Ti*CorS; and *Cr*CS (unique to *C. roseus*).^24,26,27^ Interactions between *C. roseus* DPAS and CS/TS using BiFC have been reported.^24^ These data suggest that PPIs between DPAS and various cyclases may play a key role in regulating this biosynthetic branch point. Moreover, we hypothesized that PPIs among reductase and cyclases could control the ratio of the cyclized products tabersonine, catharanthine, and 16-cmc.

**Figure 1:**
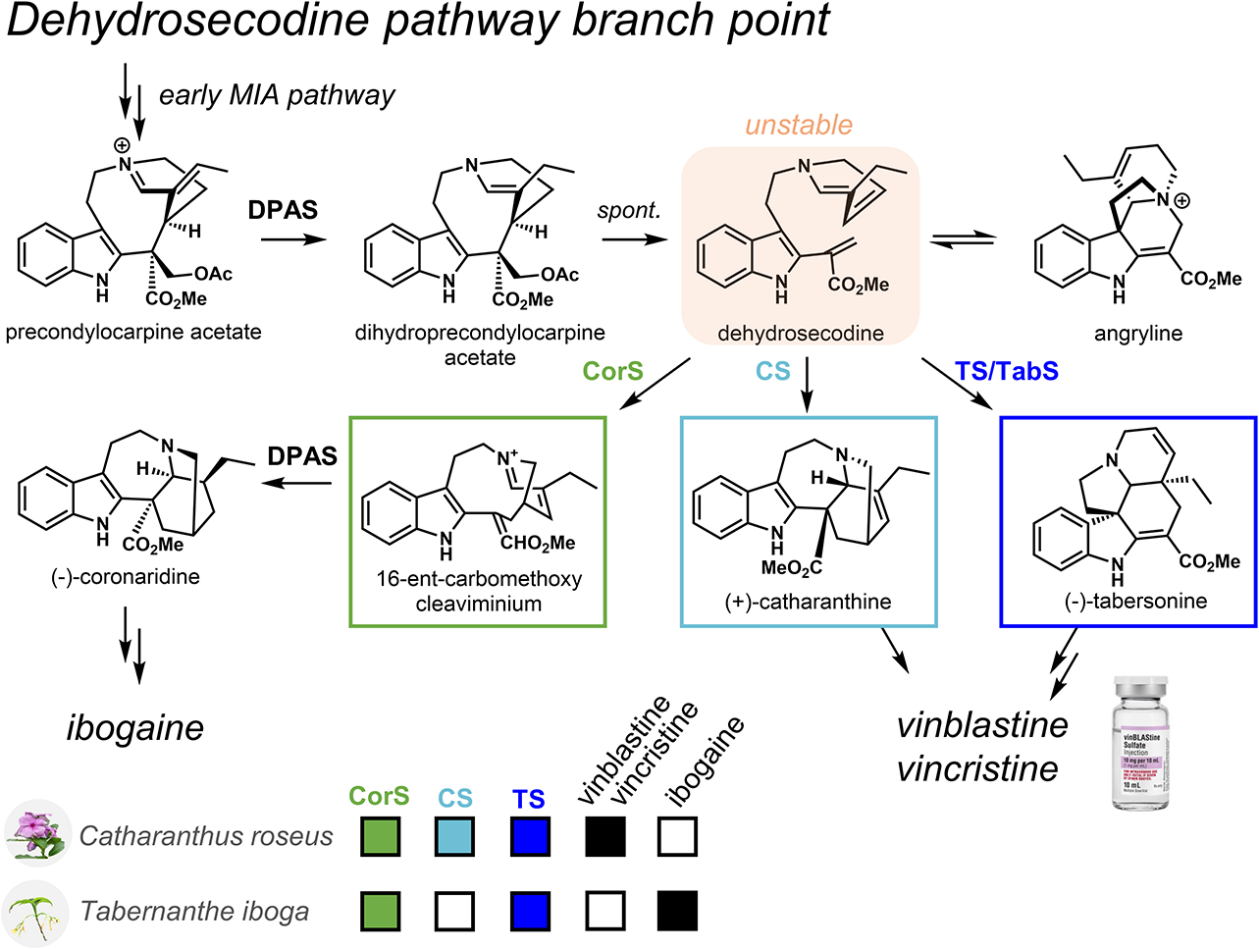
MIA pathway in *Catharanthus roseus* and *Tabernanthe iboga*. CS = catharanthine synthase, TS/TabS = tabersonine synthase, CorS = coronaridine synthase, DPAS = dihydroprecondylocarpine synthase.

Herein, we report evidence of binary interactions between DPAS and the three cyclases TS, CS, and CorS from *C. roseus* and *T. iboga*. Split-luciferase assay results support and expand upon previously obtained PPI data. We leverage these data to identify key cyclase surface residues that appear to modulate cyclase-DPAS interactions. We further demonstrate, using *in vitro* enzyme competition assays, that PPIs between DPAS and cyclases TS and CS impact the profile of the cyclization products. These results provide a glimpse into the potential role of transient PPIs in directing biosynthetic flux at this important alkaloid pathway branch point.

We first used split-luciferase assays to probe binary *C. roseus* DPAS-cyclase interactions. The split-luciferase assay is a complementation assay, leveraging the luminescence released during the luciferase-catalyzed oxidation of d-luciferin.^28^ PPIs are detected through the functional reconstitution of luciferase fragments that are tagged to a heterologously expressed protein pair of interest. Due to the catalytic nature of the d-luciferin reaction, this assay is very sensitive and can detect PPIs that are too weak to be detected by other means. As a positive control, we verified the well-characterized interaction between the *Arabidopsis thaliana* chalcone synthase (CHS) and chalcone isomerase-like (CHIL) proteins^29^ (Figure S1a). We next used this assay to demonstrate interactions between *Cr*DPAS and *Cr*TS, as well as with *Cr*DPAS and *Cr*CS. These results confirmed observations previously obtained using BiFC.^24^ Additionally, an interaction between *Cr*DPAS and *Cr*CorS was revealed by using the split-luciferase assay (Figure 2). Subcellular localization in *Nicotiana benthamiana* leaves, using N-terminal GFP and C-terminal RFP tags, demonstrated that DPAS, TS, CS, and CorS are cytosolic enzymes with varying degrees of nuclear localization that are unaffected by tagging orientation (Figure S2). We hypothesized that DPAS-cyclase PPIs would be conserved in phylogenetically related plants that also produce aspidosperma- or iboga-type MIAs. To this aim, we additionally demonstrated PPIs between DPAS/cyclase homologues from *T. iboga,* a plant that produces both aspidosperma and iboga-type MIAs through the action of *Ti*DPAS and *Ti*TabS/*Ti*CorS. Using split-luciferase assays, we showed that the two homologues of DPAS that are present in *T. iboga, Ti*DPAS1 and *Ti*DPAS2, interact with *Ti*CorS (orthologue of *Cr*CorS) and *Ti*TabS (orthologue of *Cr*TS) (Figure 2). The results of these split-luciferase assays show that PPIs between the reductase and cyclases are conserved in *T. iboga*.

**Figure 2:**
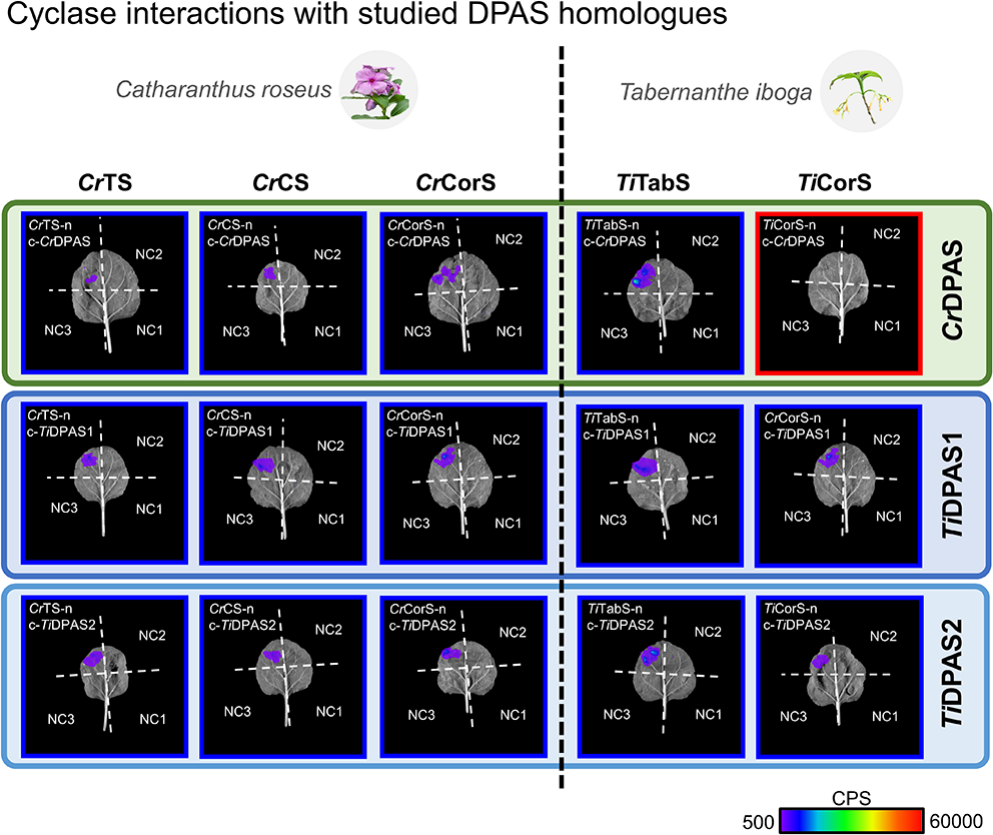
Split-luciferase assays reveal DPAS-cyclase interactions in *C. roseus* and *T. iboga*. Binary interaction between *C. roseus* and *T. iboga* DPAS and cyclases. Representative trials from three biological replicates are shown. Each leaf corresponds to the tested enzymes and three negative controls testing unfused luciferase fragments to each other (NC1) and the tested enzymes (NC2/NC3). Relative luminescence is shown on a scale of counts per second (CPS) between 500 and 60,000. Positively interacting treatments are highlighted in blue and negatives in red. Full data set showing both C and N-terminal tagged treatments is shown in Figure S1. Further validation by proteomics of representative samples is shown in Figure S3.

To probe the conservation of DPAS-cyclase interactions in *C. roseus* and *T. iboga*, cross-species DPAS-cyclase pairs were assayed: *Cr*DPAS was assayed with *T. iboga* cyclases (*Ti*TabS, *Ti*CorS), and *Ti*DPASs were assayed with *C. roseus* cyclases (*Cr*TS, *Cr*CS, *Cr*CorS). *Ti*DPAS1/2 retained interactions with all three *C. roseus* cyclases. However, *Cr*DPAS showed interaction only with *Ti*TabS and not with *Ti*CorS (Figure 2). Since *Ti*CorS appeared to specifically interact with *Ti*DPAS1/2 and not *Cr*DPAS, it seemed likely that sequence differences between *Ti*CorS and *Cr*CorS (77% identity) could reveal residues involved in the DPAS-cyclase interaction.

We hypothesized that residues on the surfaces of these proteins could contribute to the specificity of these interactions. Using the crystal structures of *Cr*TS, *Cr*CS, *Ti*CorS (PDB 6RS4, 6RT8, and 6RJ8, respectively)^30^ and models of *Cr*CorS and *Ti*TabS,^31^ we identified nonconserved surface residues between the interacting cyclases and *Ti*CorS (Figure S4a). These residues were swapped between *Cr*CorS and *Ti*CorS, leading us to generate the multisite mutants **M** ^**4**^ (*Ti*CorS: D32N/K214S/I222N/H299E; *Cr*CorS: N32D/S216K/N224I/E301H) and **M** ^**5**^ (*Ti*CorS: N24D/K26R/K72E/L165F/L279I; *Cr*CorS: D24N/R26K/E72K/F167L/I281L) for initial PPI screening. Interactions of these multisite CorS variants with the respective DPAS enzymes were then assayed using split-luciferase assays (Figure S4b). The M^5^ variant of *Cr*CorS and *Ti*CorS did not show any detectable change in the interactions with DPAS. However, the M^4^ variant displayed swapped interaction outcomes with *Cr*DPAS relative to those of the wild-type sequences. Subsequent split-luciferase assays of single mutants revealed that only position 224/222 (*Cr*CorS/*Ti*CorS numbering) was able to modulate the interaction with *Cr*DPAS for both CorS homologues. The *Cr*CorS N224I and E301H variants no longer interacted with *Cr*DPAS. Analogously, The *Ti*CorS D32N and I222N variants now showed interaction with *Cr*DPAS (Figure S4c). To investigate the significance of positions 224/222 (*Cr*CorS/*Ti*CorS numbering) for other *C. roseus* DPAS-cyclase interactions, we designed variants of *Cr*TS and *Cr*CS by replacing the native residues corresponding to position 224 of *Cr*CorS with isoleucine. All *C. roseus* cyclase Ile variants showed a loss of *Cr*DPAS interaction in split-luciferase assays (Figure 3). Consistent with *C. roseus* DPAS-cyclase interactions, Ile substitution at the equivalent position in *Ti*TabS (N222I) also disrupted interactions with *Ti*DPAS1 and *Ti*DPAS2 (Figure S5). The reciprocal substitution in *Ti*CorS (I222N) retained interaction with *Ti*DPAS1 and only disrupted interaction with *Ti*DPAS2 in one tagging orientation (Figure S5). Collectively, these results demonstrate that the amino acid at this critical position modulates DPAS-cyclase interactions in *C. roseus* and, to a lesser extent in *T. iboga*, when expressed in *N. benthamiana* leaves.

**Figure 3:**
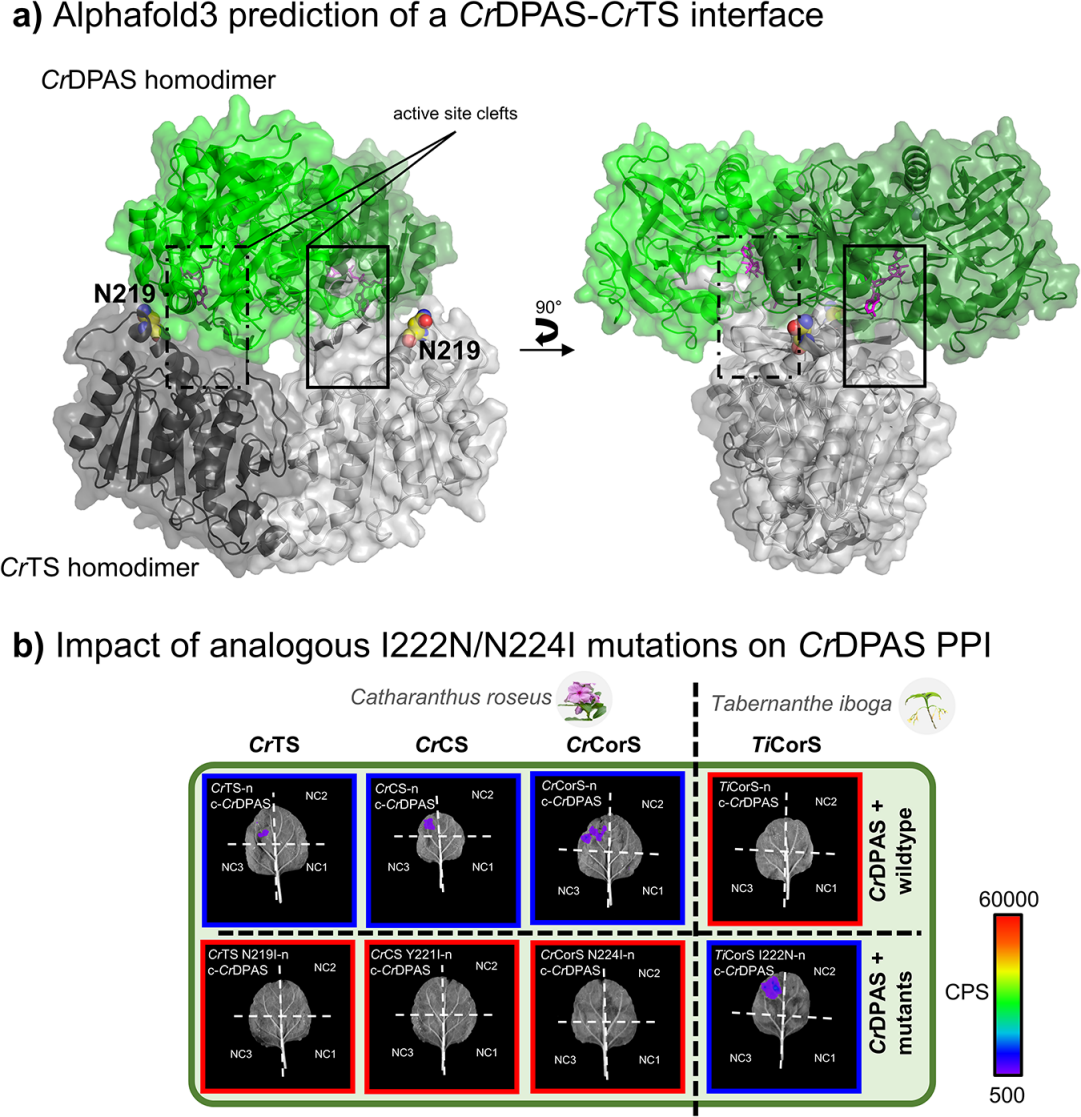
Modulation of *Cr*DPAS-cyclase interactions as revealed through mutagenesis-coupled split-luciferase assays. a) AlphaFold3 ^32^ prediction of a putative *Cr*DPAS/*Cr*TS interface. ipTM (0.48) and pTM (0.55) scores indicate limited confidence in the predicted quaternary arrangement. Therefore, the model is presented cautiously as a visualization of a *possible Cr*DPAS/*Cr*TS interface that is congruent with mutagenesis-coupled split-luciferase data. The critical residue N219 (shown in spheres) contributes to the predicted interface. Carbon atoms are colored yellow, oxygen red, and nitrogen blue. *Cr*DPAS is shown in green, *Cr*TS in gray, NADPH in magenta, and coordinated zinc ions as green spheres. *Cr*DPAS and *Cr*TS protomers are indicated by different shades of the base color. b) A single point mutation toggles DPAS-cyclase interactions. Representative trials from three biological replicates are shown. Each leaf corresponds to the tested enzymes and three negative controls testing unfused luciferase fragments to each other (NC1) and the tested enzymes (NC2/NC3). Relative luminescence is shown on a scale of counts per second (CPS). Positively interacting treatments are highlighted in blue and negatives in red.

Coronaridine appears to accumulate only in young seedlings, while in contrast catharanthine and vindoline (downstream from tabersonine) levels remain relatively constant throughout the life of the plant.^33^ Given the temporal isolation of coronaridine biosynthesis in *C. roseus*, we focused on exploring how protein interactions impact the product distribution from the *Cr*DPAS-*Cr*TS/*Cr*CS branch point. Specifically, we used a competition assay to probe how a critical mutation at positions analogous to *Cr*CorS residue 224 and shown to impact the interaction with DPAS changes the ratio of tabersonine and catharanthine products. We recreated this branch point *in vitro* via the production of dehydrosecodine from the DPAS-catalyzed reduction of PCA (Figure 4). Cyclase competition assays were conducted following the hypothesis that product distributions from *Cr*TS/*Cr*CS competitions will be impacted by mutation at this putative PPI-mediating residue. In this manner, cyclases harboring the disrupting Ile mutation were expected to be at a disadvantage compared with the wild-type DPAS-interacting cyclase. Competition assays between wild-type *Cr*TS and *Cr*CS Y213I resulted in a product profile skewed more toward the TS product, tabersonine. Conversely, competition assays between wild-type *Cr*CS and *Cr*TS N219I resulted in an increase in the concentration of the CS product, catharanthine. Competition assays of *Cr*TS N219I and *Cr*CS Y213I resulted in a return to the wild-type *Cr*TS/CS assay ratio. Notably, the TS variant was no less active than the wild-type cyclase, as demonstrated by the total amount of product formed from competition reactions (Figure S6) and single enzyme reactions (Figure S7). *Cr*CS was not included in Figure S7 because an impurity that overlaps with catharanthine prevents accurate product quantification.^24^ Overall, these results suggest that disruption of *Cr*DPAS-*Cr*TS/*Cr*CS interactions, observed through split-luciferase assays, likely contribute to altering product profiles during *in vitro* competition assays.

**Figure 4:**
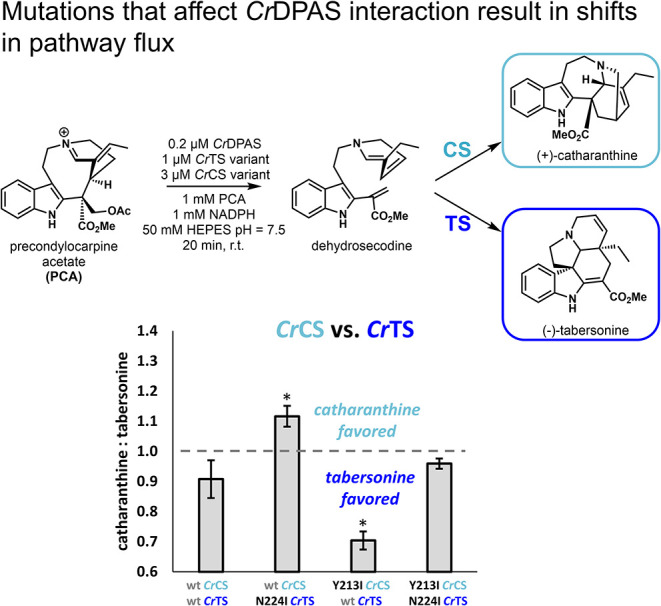
Results from *in vitro* competition reactions of precondylocarpine acetate (PCA) with *Cr*CS variants and *Cr*TS variants. The resultant ratio of catharanthine to tabersonine is plotted for each competing enzyme pair and is reported as an average of technical triplicates. * = Student’s t test <0.05 from wild-type (wt) condition. See Figure S6 for additional analysis.

We attempted to further characterize *C. roseus* DPAS-cyclase interactions by FRET-FLIM using eGFP and mRFP1 protein fusions expressed in *N. benthamiana* leaves. An interaction between fluorophore-tagged proteins that bring GFP and RFP within 10 nm of each other facilitates energy transfer (FRET) from excited GFP to RFP, subsequently decreasing the fluorescence lifetime of GFP.^34^ Despite the detection of FRET in our positive control, between *At*CHS and *At*CHIL, no FRET was detected between *Cr*DPAS and any of the *Cr*TS, *Cr*CS, or *Cr*CorS (Figure S8). Although we have evidence of DPAS-cyclase interactions using split-luciferase, *in vitro* competition, and BiFC assays,^24^ the lack of FRET signal suggests that these proteins interact weakly or transiently. Many of our interactions detected through split-luciferase assays are at the lower-limit of detection, which further supports transiency in these PPIs.

Here we show, using binary split-luciferase assays, that DPAS interacts with three downstream cyclases when expressed in *N. benthamiana* leaves. We used competition assays to probe these putative interactions *in vitro* and demonstrated that *in planta* interaction-disrupting mutations of cyclases also impact the flux of substrates into two distinct cyclized products *in vitro*. The low intensity of split-luciferase assays and failure to detect the FRET signal in *N. benthamiana* highlight that DPAS-cyclase PPIs are likely transient or weak. Nevertheless, based on these findings, we hypothesize that transient PPIs between DPAS and downstream cyclases mediate the dehydrosecodine branch point during the biosynthesis of tabersonine and catharanthine, both precursors to the commercially valuable bisindole alkaloids vincristine and vinblastine. In an early study on flavonoid biosynthetic PPIs, competition for interaction with *At*CHS between flavanone 3-hydroxylase (F3H1) and dihydroflavonol 4-reductase (DFR) was proposed to mediate flux through respective branches.^6^ This concept has been greatly expanded over the years, more broadly, in phenylpropanoid biosynthesis. More recently, distinct modules mediating biosynthetic branches of flavonoids in *Camellia sinensis* were proposed based on PPIs between flavonoid biosynthetic enzymes (including CHS, F3H, and CFR).^17^ In another example, PPIs between three maize (*Zea mays*) indole-3-glycerolphosphate synthases with downstream lyases were proposed to contribute to the modulation of indole pools for tryptophan, benzoxazinoids, and free indole biosynthesis.^18^ It is becoming increasingly clear that transient or weak protein interactions play crucial roles in mediating specificity in metabolic pathways. Our findings provide a glimpse into the likely dynamic orchestration of MIA biosynthetic enzymes and represent a small step into the largely unexplored organization of this biosynthetic pathway.

## Supplementary Material



## Notes

Supplemental figures, materials and methods, associated DNA and protein sequences, and experimental details (PDF)

## Abbreviations

PPIs: Protein–protein interactions
BiFC: Biomolecular Fluorescence Complementation
FRET: Förster Resonance Energy Transfer
FLIM: Fluorescence Lifetime Imaging Microscopy
MIA: Monoterpene Indole Alkaloid
DPAS: Dihydroprecondylocarpine Acetate Synthase
PCA: Precondylocarpine Acetate (PCA)
TS or TabS: Tabersonine Synthase
CS: Catharanthine Synthase
CorS: Coronaridine Synthase
16-cmc: 16-ent-carbomethoxycleaviminium
