# Supplementary material for: Protein–Protein Interactions Modulate a Key Branch Point in Monoterpene Indole Alkaloid Biosynthesis
Source: ACS Chem Biol. 2026 Jan 4;21(1):8–13. doi: 10.1021/acschembio.5c00485 (PMC12813968; doi:10.1021/acschembio.5c00485)
Supplement: Supplementary file 1 [file cb5c00485_si_001.pdf]

Supplementary Information

Protein-Protein Interactions Modulate a Key Branch Point Intermediate in Monoterpene Indole Alkaloid Biosynthesis

Authors: Samuel C. Carr[‡], Allwin McDonald[‡], Chloe Langley[‡], Veit Grabe, Klaus Gase, Sarah E. O'Connor[*]

[‡]These authors contributed equally
*Correspondence to: oconnor@ice.mpg.de

Contents:

Supplementary Figures S2– S9

Materials and Methods S10– S19

Protein and DNA sequences S19– S24

Supplementary References S24

Supplementary Figures

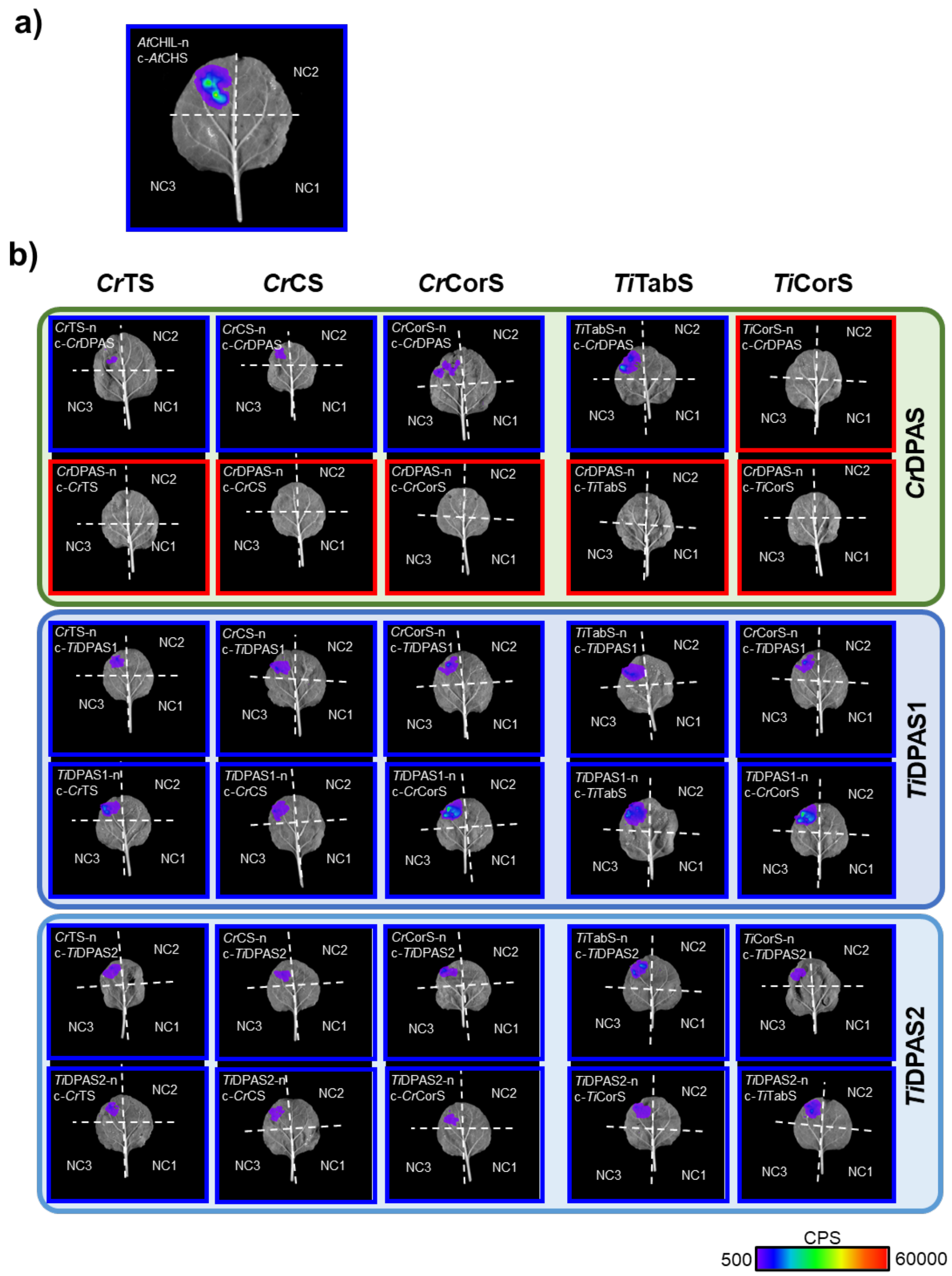

Figure S1: a) Representative trial from a split-luciferase assay of three biological replicates for a positive control of interaction between *Arabidopsis thaliana* chalcone isomerase-like (CHIL) and chalcone synthase (CHS)[1]. Each leaf corresponds to the tested enzyme pair and three negative controls testing unfused luciferase fragments to each other (NC1) and the tested enzymes (NC2/NC3). **b)** Representative trials from a split-luciferase assay of three biological replicates for each tested enzyme pair to test for interaction between various cyclases (*Cr*TS, *Cr*CS, *Cr*CorS, *Ti*TabS, *Ti*CorS) and dihydroprecondylocarpine acetate synthases (DPAS). Blue outlines indicate an observed interaction, and red outlines indicate no observed interaction. '-n' = C-terminally-tagged N-terminal luciferase fragment, 'c-' = N-terminally-tagged C-terminal luciferase fragment, *Cr* = *Catharanthus roseus*, *Ti* = *Tabernanthe iboga,* CS = catharanthine synthase, TS/TabS = tabersonine synthase, CorS = coronaridine synthase. Proteomic analysis of the *At*CHIL-*At*CHS positive control and both tagging orientations of *Cr*DPAS-*Cr*TS reveal that the proteins of interest were present in the respective samples, with the exception of *Cr*DPAS-nLuc (Figure S3). The absence of *Cr*DPAS-nLuc specific peptides suggests that the lack of split-luciferase signal in all *Cr*DPAS-nLuc assays may be due to weak or absent expression and not loss of PPI.

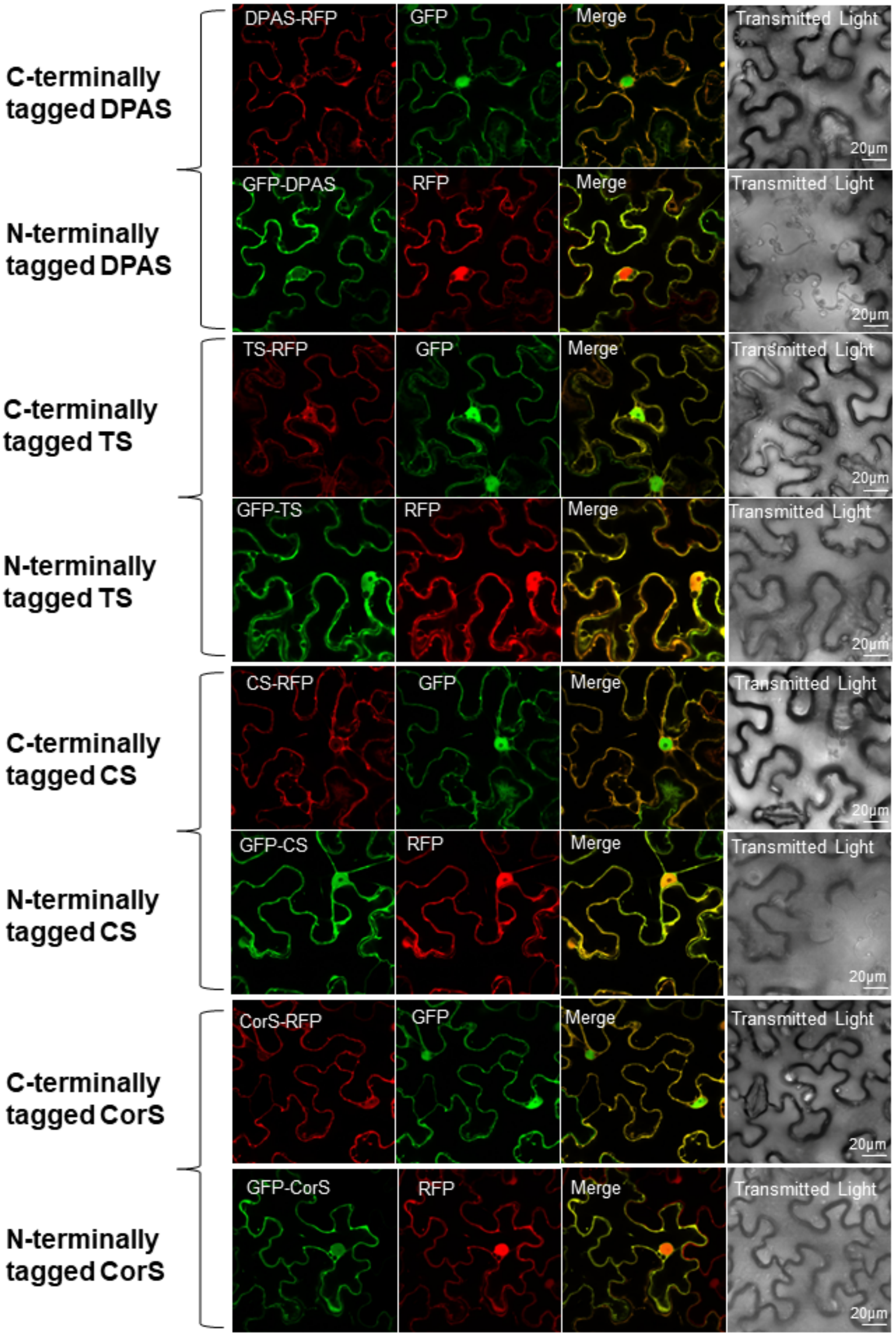

Figure S2: Subcellular localization of *C. roseus* DPAS, TS, CS, and CorS in *N. benthamiana* leaves. C-terminal RFP and N-terminal GFP tags were used to localize the proteins of interest and free RFP or GFP were used as cytosolic/nuclear markers. Green corresponds to GFP, red to RFP, yellow to merged GFP-RFP, and grey scale for transmitted light.

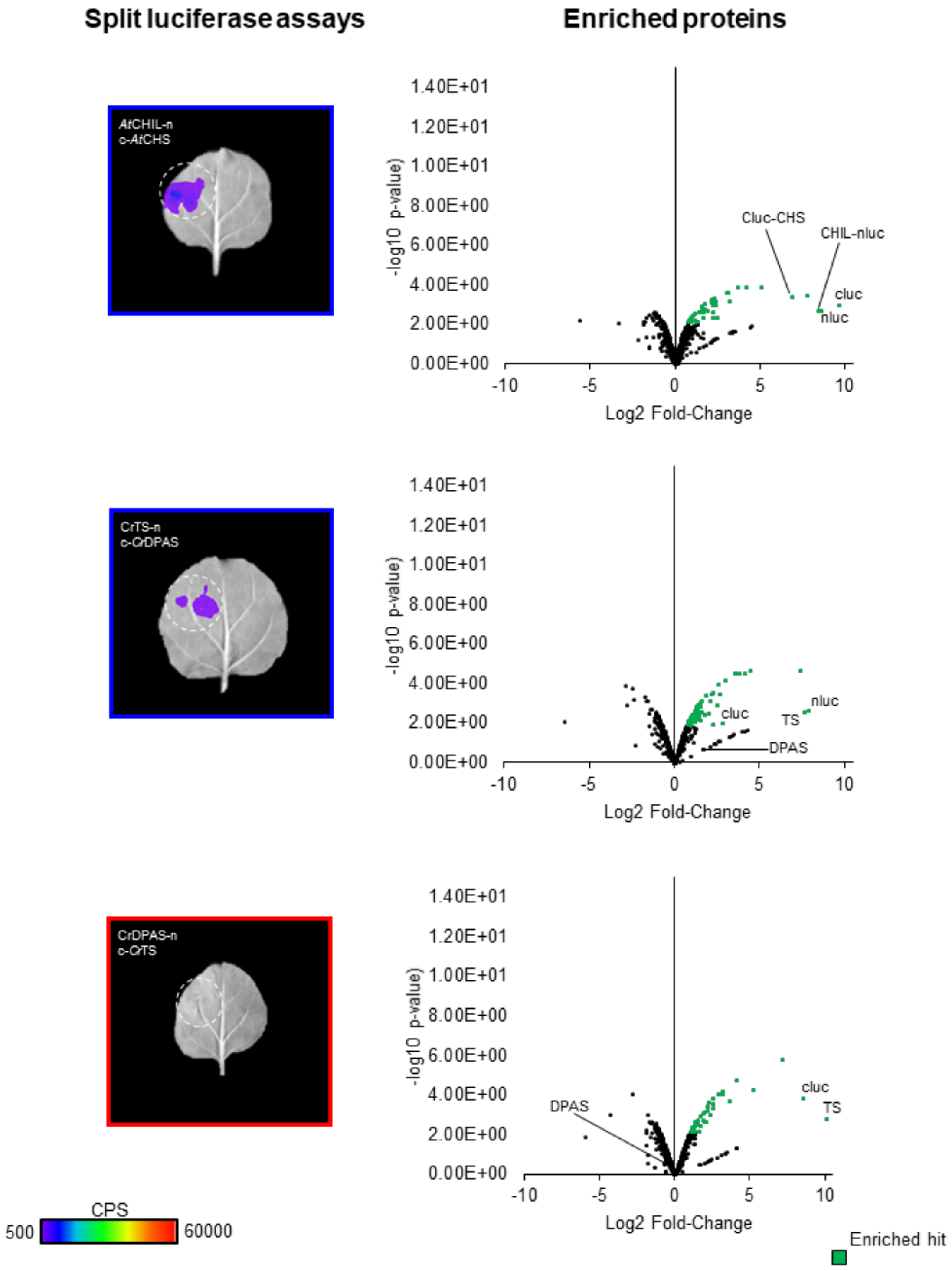

Figure S3: Proteomics analysis of representative split-luciferase assays. Split-luciferase assay results are shown on the left and the corresponding volcano plot from proteomics on the right. Enriched peptides compared to untransformed *N. benthamiana* leaves are colored green and peptides corresponding to proteins of interest are labeled. Proteomics data was collected from two biological replicates. Infiltrated regions of the leaves are circled, and the respective assay pair is marked above the leave.

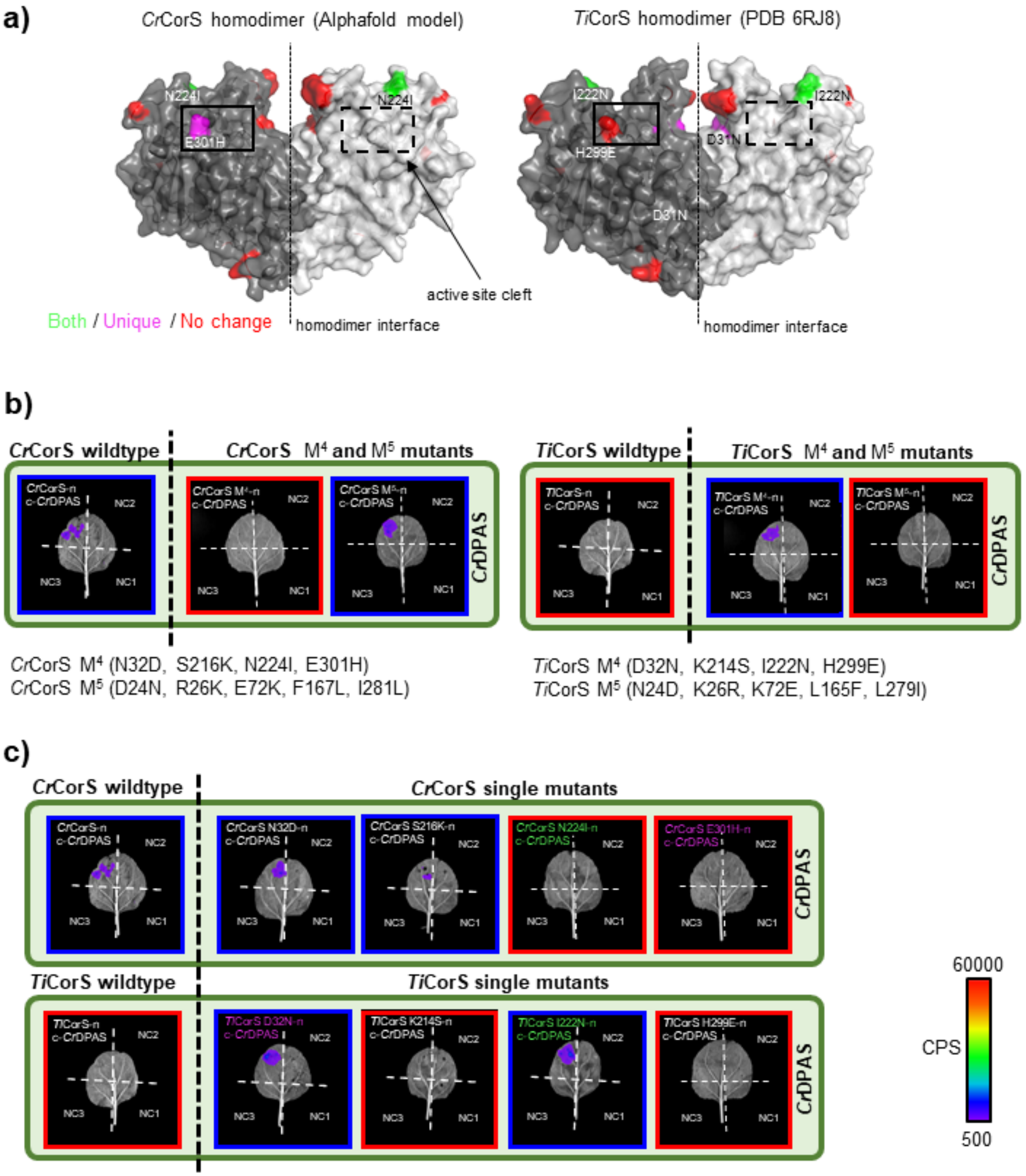

Figure S4: Structural analyses of *Cr*CorS and *Ti*CorS, and representative trials from split-luciferase assays of three biological replicates. **a)** Location of CorS mutated surface residues on *Cr*CorS (Alphafold model) and *Ti*CorS (PDB 6RJ8). Protomers of the conserved cyclase dimer are shown in dark and light shades of grey. Red indicates residues that did not alter interactions with *Cr*DPAS, magenta indicates residues that altered interactions in only one of *Cr*CorS or *Ti*CorS, and green indicates residues that altered interactions in both *Cr*CorS and *Ti*CorS. The active site cleft is marked by blue rectangles, and the dotted lines indicate that the cleft is on the opposite side of the enzyme. b**)** Representative trials from split-luciferase assays testing the interactions of the multisite M^4 and M^5 variants of *Cr*CorS and *Ti*CorS with *Cr*DPAS. **c)** Representative trials from split-luciferase assays testing the single substitution from the M^4 variants. Blue outlines indicate an observed interaction, and red outlines indicate no observed interaction. '-n' = C-terminally-tagged N-terminal luciferase fragment, 'c-' = N-terminally-tagged C-terminal luciferase fragment.

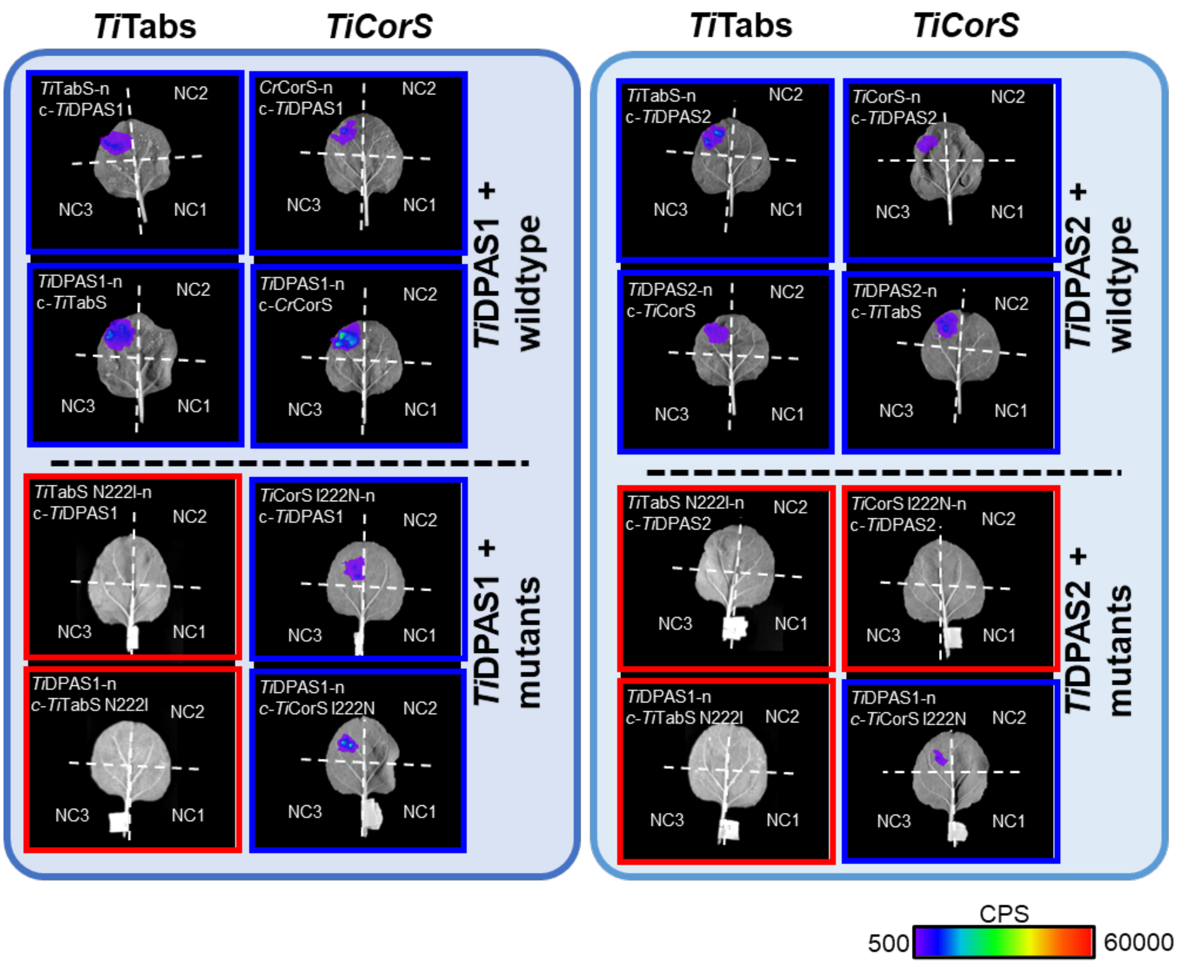

Figure S5: Representative trials from a split-luciferase assay of three biological replicates for *Ti*TabS and *Ti*CorS single mutation variants' interactions with TiDPAS1/2. Each leaf corresponds to the tested enzyme pair and three negative controls testing unfused luciferase fragments to each other (NC1) and the tested enzymes (NC2/NC3). Blue outlines indicate an observed interaction, and red outlines indicate no observed interaction. '-n' = C-terminally-tagged N-terminal luciferase fragment, 'c-' = N-terminally-tagged C-terminal luciferase fragment.

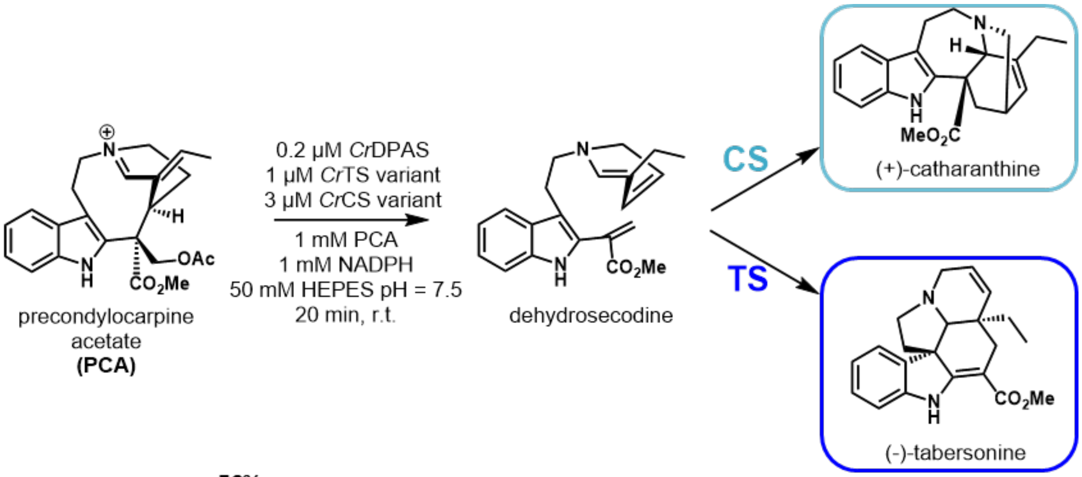

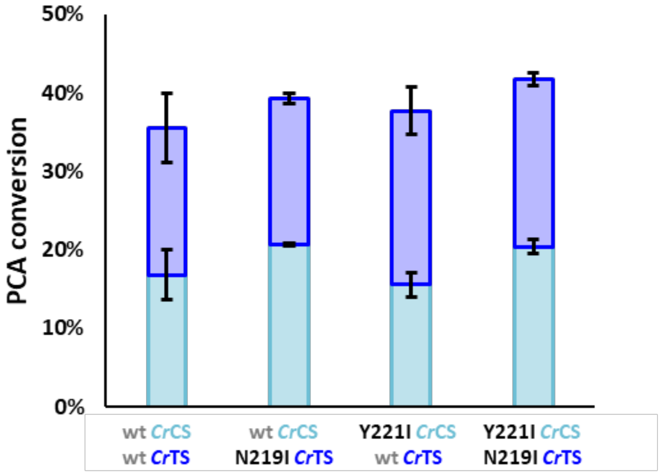

Figure S6: Results from *in vitro* competition reactions of precondylocarpine acetate (PCA) with *Cr*CS variants and *Cr*TS variants. The data presented here is the same as depicted in Figure 4, but with total PCA conversion shown. Conversion of PCA to either tabersonine or catharanthine is reported as an average of technical triplicates.

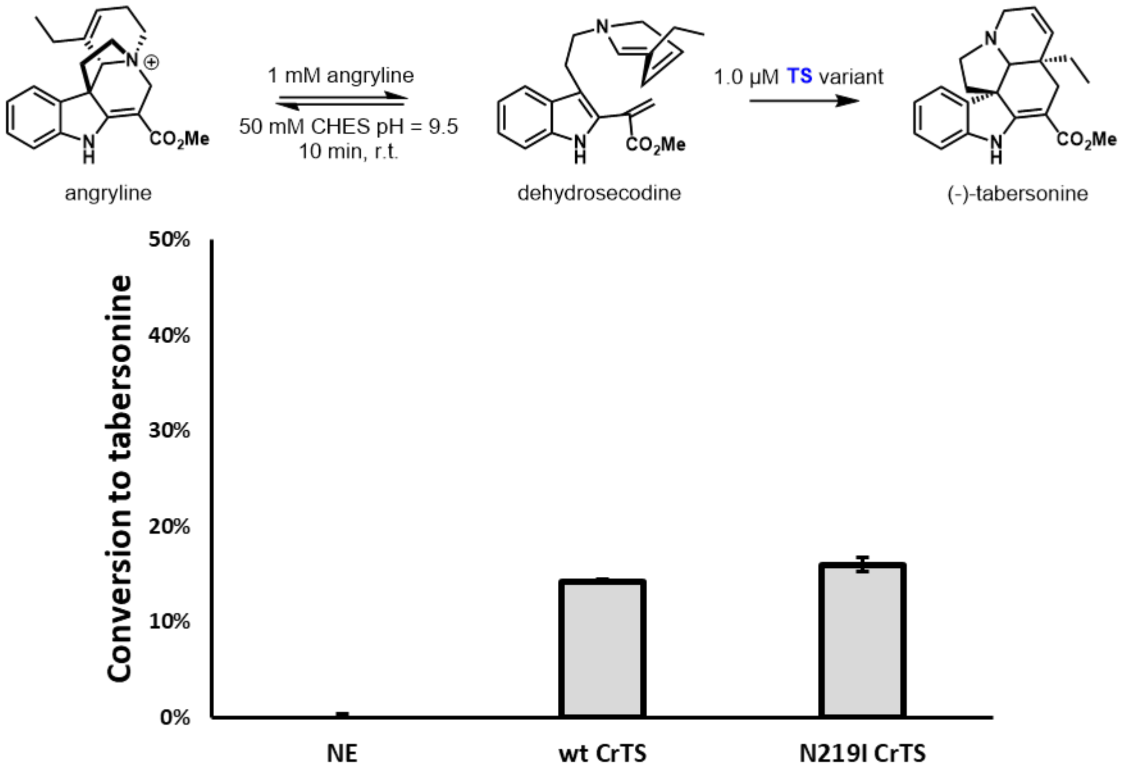

Figure S7: Results from *in vitro* reactions of no enzyme (NE), wild-type (wt) *Cr*TS, and N244I *Cr*TS with angryline. Conversion to tabersonine is reported as an average of technical quadruplicates. **Reactions with CrCS variants were performed, but due to an impurity from the angryline substrate with the same m/z and near-identical retention time, analysis of the results was not fruitful. See Caputi et al. 2018*[2] *for more information.*

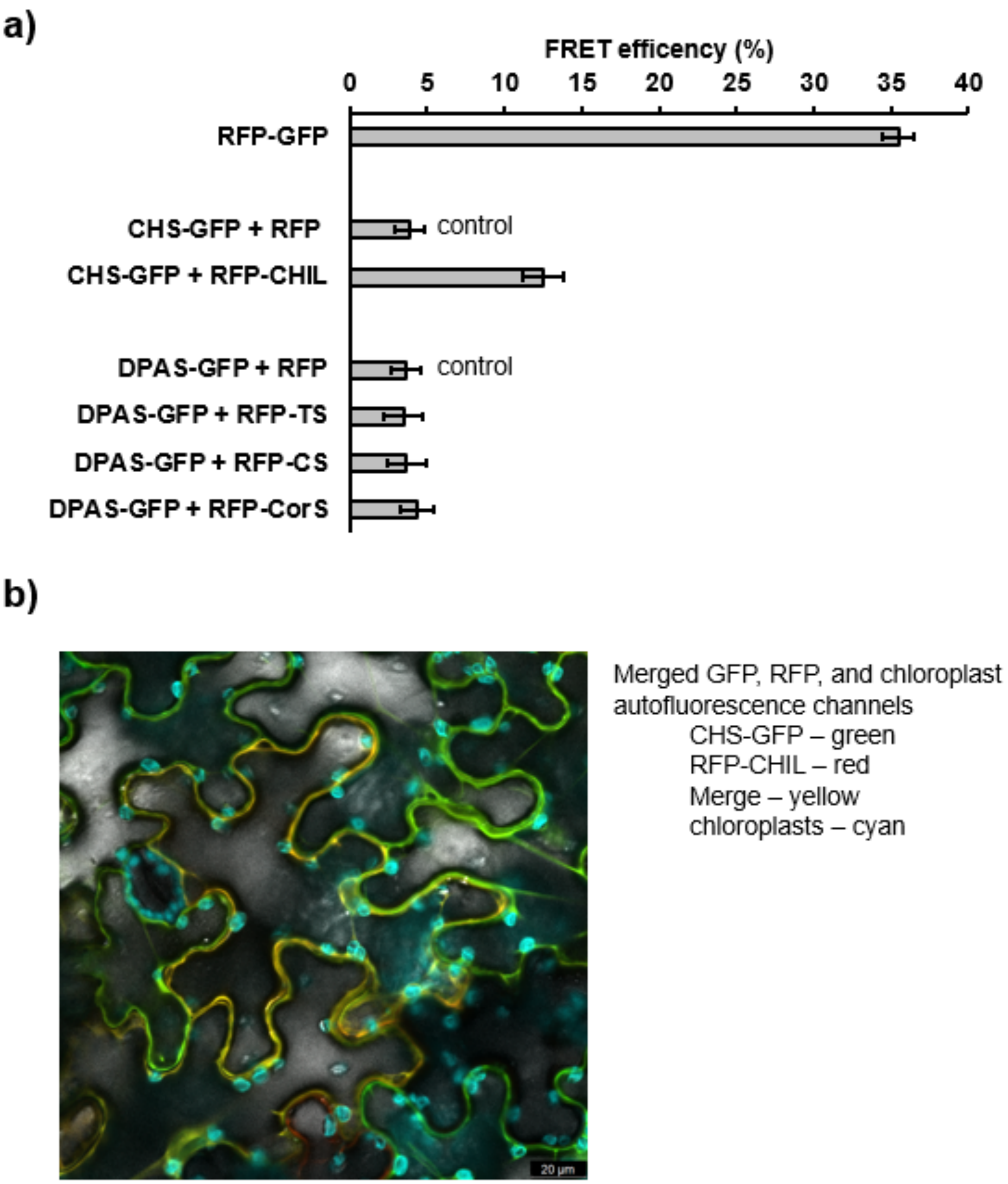

Figure S8: FLIM-FRET of *Catharanthus roseus* DPAS with the cyclases CS, TS, and CorS. a) FRET efficiencies of DPAS-cyclase pairs. A fusion of RFP and GFP was used as a control and the previously reported *Arabidopsis thaliana* CHS and CHIL interacting pair[1] was used as a positive protein-protein interaction control. Data represents the average of six FRET efficiencies calculated from GFP fluorescence lifetimes from six images representing three biological replicates. b) Merge view of CHS-GFP (green), CHIL-RFP (red), and chlorophyl autofluorescence (cyan). Colocalized GFP and RFP fluorescence is shown in yellow, and the scale bar indicates 20 µm

Materials and Methods

Plants and Plant Growth

Nicotiana benthamiana and *Catharanthus roseus* plants were grown on a standard soil mix in the greenhouse. Culture conditions were set to 22 °C, 60% relative humidity and followed a 16 hour light/8 hour dark photoperiod. Tobacco plants were grown for at least 3 weeks but no longer than four weeks prior to infiltration with *Agrobacterium tumefaciens* GV3101. Plant watering was performed periodically as needed.

Chemicals

All chemicals used in this study were purchased as molecular biology grade or higher from commercial vendors (*Sigma Aldrich*, *Thermo Fischer*, etc.) unless denoted different. Angryline was obtained from Omar Kamileen[3] and precondylocarpine acetate (PCA) was directly purified from *Catharanthus roseus* plants (see below).

Molecular biology kits

All molecular biology kits were used according to the manufacturer´s instructions, unless specified. For genes or gene fragments destined for downstream applications Platinum SuperFi II polymerase (*ThermoFisher*) was used for amplification. Gene fragments were purified by agarose gel electrophoresis (1% agarose; 120 V, 40 min) and extracted from the gel using a Zymoclean™ Gel DNA Recovery Kit (*Zymo*). All oligonucleotide primers were synthesized by and obtained from *Sigma Aldrich*. Gene cloning was routinely performed using an In-Fusion kit (*Takara*). Plasmid DNA was isolated from bacterial cultures using the Wizard® *Plus* SV Minipreps DNA Purification System kit (*Promega*).

VIGS of *Catharanthus roseus* for PCA isolation

Construction of the VIGS vector pTRV2-M129267 and VIGS followed the protocol described in Li *et al.* (2023)[4]. In brief, a 274 bp fragment of the C. roseus dihydroprecondylocarpine synthase (DPAS) gene (transcript CRO_T129267 in https://datadryad.org/stash/dataset/doi:10.5061/dryad.08vv50n) was PCR amplified from cDNA using oligonucleotides 129267B 5'-ATATTGCTGCGGATCCGAGTTGCCACCTATTCCTTT-3' and 129267X 5'-ATGCCCGGGCCTCGAGCAGAGTACACACTTATGACTTTT-3' with In-Fusion cloning overhangs. The DPAS VIGS target sequence was taken from (*Caputi et al. 2018*)[2] and comprised 240 bp of the coding region and 34 bp of the 3' UTR. In-Fusion was used to clone

the obtained PCR fragment in the *Bam*HI and *Xho*I digested VIGS vector pTRV2-MgChl [pTRV2-*ChlH*(F1R2) from (*Liscombe et al. 2011*),[5] yielding pTRV2-M129267 (map and sequence in supplemental material).

Agrobacterium tumefaciens GV3101 was transformed with pTRV2-M129267 and used for the VIGS inoculations of *C. roseus*. For this, *A. tumefaciens* GV3101 carrying the plasmid pTRV1 (https://doi.org/10.1046/j.1365-313X.2002.01297.x) and *A. tumefaciens* GV3101 (pTRV2-M129267) were grown overnight in a rotary shaker at 28 °C and 300 rpm in each 3 mL of lysogeny broth (LB) medium containing 50 µg/mL kanamycin, 25 µg/mL gentamicin and 100 µg/mL rifampicin to an OD_{600} of ~1.5. Cells were pelleted for 10 minutes at 3000 rpm and resuspended in infiltration buffer (100 µM acetosyringone, 10 mM NaCl and 1.75 mM $CaCl_2$) to an OD_{600} of 2. The cells were incubated for 2 hours on a rotary shaker at room temperature and 60 rpm, after this 450 µL of each bacterial strain were mixed. VIGS inoculation was performed by pipetting a 10 µL drop of the mixed bacterial suspension between the stem and the petiole of one leaf directly above the cotyledons of a 30 days old *Catharanthus roseus* cultivar 'Sunstorm Apricot' plant. Using a 0.40 x 25 mm needle, the stem was pierced once through the bacterial suspension drop. After this, plants were grown under *C. roseus* standard conditions. First VIGS symptoms (yellowing of the leaves due to magnesium chelatase subunit H gene silencing) were visible after 12 days. The tissue with complete gene silencing was harvested three weeks after inoculation.

PCA purification using preparative HPLC

Fresh (yellow-colored) leaves (52 g) were blended in ~300 mL MeOH until homogenous. The resultant slurry was filtered through a Buchner funnel and leaves washed 3x with 100 mL MeOH. The MeOH eluent was evaporated in a rotary evaporator and the extract resuspended in 70% MeOH. This solution was flashed through a 10 g C18 SPE cartridge and the flow-through evaporated to obtain a reddish-brown viscous liquid. This mixture was injected onto an HPLC system (Agilent 1260 Infinity II) equipped with a Phenomenex LC column (Luna® 5 µm C18 (2) 100A, 250 x 30 mm, AXIA™ Packed, Ea) with a multiple wavelength detector and fraction collector. As mobile phases A (water + 0.1 % formic acid) and B (acetonitrile) were used. The flow rate was set to 30 mL/min and the gradient was as follows: 10 % B for 1 min, 10 -> 30% B from 1-19 min, 30 -> 100% B from 20-21 min, 100% B from 21-25 min, 100 -

> 10% B for 25-26 min, and 10% B from 26-30 min. PCA eluted around 15 min, and peaks containing PCA were collected, pooled, and evaporated. These concentrated fractions were rerun with the same above method to obtain purer compound. These fractions were again pooled and evaporated to obtain 10 mg of a yellow powder comprising ~ 80% PCA (as verified by an authentic standard).

Cloning and mutagenesis

Full-length *C. roseus* DPAS, TS, CS, CorS and *T. iboga* DPAS1/2, TabS, CorS genes were amplified from leaf cDNA as described previously[3,6] with correct overhangs for pCambia nLuc, pCambia cLuc, pOPINF, pCambia eGFP, or pCambia mRFP1 vectors. *Cr*TS, *Cr*CS, *Cr*CorS, *Ti*TabS, and *Ti*CorS Mutants were generated using overlap extension PCR. Gel purified PCR products were ligated into digested pCambia or pOPINF vectors by In-Fusion. pCambia nLuc/eGFP vectors were digested with *KpnI* and *SalI*, pCambia cLuc/mRFP1 with *KpnI* and *PstI*, and pOPINF with *HindIII* and *KpnI*. In-Fusion assemblies were transformed into *Escherichia coli* Top10 competent cells (*ThermoFisher*) by heat shock at 42 °C, plated on LB agar supplemented with 100 µg/mL kanamycin (pCambia) or 100 µg/mL carbenicillin (pOPINF), and grown overnight at 37 °C. Single colonies were used to inoculate overnight cultures supplemented with the correct antibiotic and plasmids were isolated and sequence verified.

Transient gene expression in *Nicotiana benthamiana*

Electrocompetent cells of *Agrobacterium tumefaciens* GV3101 (*Goldbio*) were thawed on ice and mixed with plasmid DNA (vector = pCambia, ~500 ng) that had been sequence-verified by Sanger sequencing. Upon thawing, the cell suspension was transferred to pre-chilled electroporation cuvette and cells were electroporated using a MicroPulser™ (*BioRad*) at 2.2 kV. Cells were mixed with 0.6 mL LB medium and recovered at 28 °C/225 rpm for 3 hours prior to plating on selective LB agar plates (supplemented with 20 µg/mL rifampicin, 50 µg/mL gentamycin and 50 µg/mL kanamycin). Plates were kept at 28 °C for 2 days. Single colonies were used to inoculate liquid cultures. Liquid cultures were prepared as 5 mL cultures (supplemented with 20 µg/mL rifampicin, 50 µg/mL gentamycin and 50 µg/mL kanamycin) and cultivated at 28 °C and 250 rpm for up to 24 hours. 25 % glycerol stocks were prepared thereof, snap frozen in liquid nitrogen and stored at –70 °C indefinitely. The remaining culture was then used for transient expression in *Nicotiana benthamiana*. Cells were harvested by centrifugation

at 4000 x g for 10 minutes and the pellet gently resuspended in infiltration buffer (10 mM MES, 10 mM $MgCl_2$, pH 5.6, 200 μM acetosyringone) to OD_{600} = 0.6. Resuspended cultures were incubated with gentle shaking in the dark for 3 hours. Cultures were mixed 1:1 with the additional strains so each strain was infiltrated at a final concentration of OD_{600} = 0.3. Strains were infiltrated in the abaxial side of a 3–4-week-old *N. benthamiana* leaf from the 2nd fully expanded leaf pair.

Split-luciferase Complementation Assays

N. benthamiana leaves overexpressing nLuc or cLuc pCambia constructs were imaged 3 days post infiltration using a NightShade LB 985 (*Berthold Technologies*). Leaves were divided into four quadrants consisting of the enzyme-pair in question (top left) and three negative controls (top right, bottom left, and bottom right). Each treatment was infiltrated as a spot within the confines of the quadrant. Leaves were sprayed with 0.5 mM solution of D-luciferin and incubated in the dark for 5 minutes before being imaged on their abaxial side. Images were exposed for 0.1 seconds, and luminescence emission exposed for 20 seconds with 8 x 8-pixel binning. Up to four leaves of the same combination were imaged per experiment until three leaves gave the same result, and each experiment was repeated an additional time. The previously published *At*CHIL-nLuc and *At*CHS-cLuc combination was used as a positive control in each infiltrated batch (Figure S1a)[1].

Proteomics

A subset of three split-luciferase pairs were selected for validation using proteomics: cLuc-*At*CHS + *At*CHIL-nLuc, cLuc-*Cr*DPAS + *Cr*TS-nLuc, and *Cr*DPAS-nLuc + cLuc-*Cr*TS. 2 g of *N. benthamiana* leaves expressing assay pairs were ground with a mortar and pestle in liquid nitrogen. These were then incubated in 30 mL of buffer (50 mM TRIS-HCl pH 8.0, 50 mM glycine, 500 mM NaCl, 20 mM imidazole, 5% (v/v) glycerol) for 2 hours at 4 °C with gentle shaking. Samples were centrifuged at 4000 x g for 10 minutes at 4 °C and the supernatant collected. NaCl concentration was reduced to 100 mM using 10 kDa molecular weight cut-off centrifugal filter (*Merck*) then samples were concentrated to 1mg/mL. Concentration was determined using a Bradford Assay (Thermo Scientific) and 20 μg of protein sent to Proteomics core Facility, EMBL (Heidelbarg, Germany) for proteomic analysis. Methodology reported by EMBL is shown below:

Sample preparation – Protein samples were subjected to the SP3 protocol (PMID 25358341) conducted on the KingFisher Apex™ platform (Thermo Fisher). For digestion, trypsin was used in a 1:20 ratio (protease:protein) in 50 mM Triethylammonium bicarbonate (TEAB) supplemented with 5 mM Tris(2-carboxyethyl)phosphine hydrochloride (TCEP) and 20 mM 2-chloroacetamide (CAA). Digestion was carried out for 5 hours at 37°C. Peptides were dried down and taken up in 4% acetonitrile and 1% formic acid prior to LC-MS/MS analysis.

MS measurement – An UltiMate 3000 RSLCnano LC system (Thermo Fisher Scientific) equipped with a trapping cartridge (µ-Precolumn C18 PepMap™ 100, 300 µm i.d. × 5 mm, 5 µm particle size, 100 Å pore size; Thermo Fisher Scientific) and an analytical column (nanoEase™ M/Z HSS T3, 75 µm i.d. × 250 mm, 1.8 µm particle size, 100 Å pore size; Waters). Samples were trapped at a constant flow rate of 30 µL/min using 0.05% trifluoroacetic acid (TFA) in water for 6 minutes. After switching in-line with the analytical column, which was pre-equilibrated with solvent A (3% dimethyl sulfoxide [DMSO], 0.1% formic acid in water), the peptides were eluted at a constant flow rate of 0.3 µL/min using a gradient of increasing solvent B concentration (3% DMSO, 0.1% formic acid in acetonitrile). The outlet of the analytical column was directly coupled to an Orbitrap Exploris™ 480 mass spectrometer (Thermo Fisher Scientific) equipped with a Nanospray Flex™ ion source, operating in positive ion mode. Peptides were introduced into the instrument using a Pico-Tip emitter (360 µm OD × 20 µm ID; 10 µm tip, CoAnn Technologies) with a spray voltage of 2.5 kV. The capillary temperature was maintained at 275 °C. Full MS (MS1) scans were acquired in centroid mode over an m/z range of 420–680, with a resolution of 60,000 at m/z 200 in the Orbitrap. The automatic gain control (AGC) target was set to 'custom' with a normalized AGC target of 300%. Data-independent acquisition (DIA) was performed across a precursor mass range of 430–670 m/z, using 4 m/z isolation windows. MS/MS scans were acquired in centroid mode over a scan range of 200–1800 m/z, with a resolution of 30,000. The AGC target for DIA was set to 'custom', with a normalized value of 3000%. A normalized higher-energy collisional dissociation (HCD) energy of 28% was applied for fragmentation.

Database search – Raw files were analyzed using DIA-NN 1.8.1 (PMID: 31768060) with directDIA module using an in silico DIA-NN predicted spectral library (Trypsin as protease; C carbamidomethylation and N-terminal M excision; Protein N-terminal acetylation and Oxidation

on Methionine enabled; 2 missed cleavages; precursor m/z range: 430-670; precursor charge range 2 to 4). The spectral library was generated from fasta files downloaded from Uniprot (Nicotiana Benthamia, 08.05.2023, 925 entries), common contaminants, and the protein sequences of DPAS-nLuc, cLuc-DPAS, TS-nLuc, cLuc-TS, CHIL-nLuc, and cLuc-CHS. The DIA-NN search used the following parameters: Precursor FDR (%)= 1; Mass accuracy, MS1 accuracy, and Scan window = 0; Use isotopologues; match between runs enabled; No shared spectra; Protein inference: Genes, Neural network classifier: Single-pass mode; Quantification strategy: Robust LC (high precision); Cross-run normalization: RT-dependent; Library generation: Smart profiling; Speed and RAM usage: optimal results.

Data processing – DIA-NN output (report.tsv) was processed to generate filtered results at the protein level for further downstream analysis. To ensure high data quality, the following filtering criteria were applied to the DIA-NN results: Q.Value <= 0.01, PG.Q.Value <= 0.01, and Lib.PG.Q.Value <= 0.01. Functions from the diann-rpackage (https://github.com/vdemichev/diann-rpackage/blob/master/R/diann-R.R) were employed to load, aggregate, and quantify proteins using DIA-NN's MaxLFQ algorithm (PMID: 31768060). For proteins lacking gene annotations, the 'Protein.ID' was used to fill in missing 'Gene' entries, ensuring comprehensive gene-level representation. Also the 'PG.MaxLFQ' quantity was used in these cases to fill the missing 'Genes.MaxLFQ.Unique' entries. The filtered table, 'diann_outputs_filtered_report.uniqueGenes_maxLFQ_matrix.tsv', was generated based on the 'Genes.MaxLFQ.Unique' column of the DiaNN report output. This matrix includes protein-level data, enriched with protein-level summaries, such as the number of Razor Peptides, Unique Peptides, Total Peptides, and the Total Intensity, which were calculated on the combined dataset and integrated into the gene-level output. The quantification is only based on proteotypic peptides. The filtered table, 'diann_outputs_filtered_report.ProteinGroups_maxLFQ_matrix.tsv', was generated based on the 'PG.MaxLFQ' column of the DiaNN report output. This matrix includes protein-level data, enriched with protein-level summaries, such as the number of Razor Peptides, Unique Peptides, Total Peptides, and the Total Intensity, which were calculated on the combined dataset and integrated into the gene-level output. The quantification is based on all peptides.

Subcellular localization

The subcellular localization of *C. roseus* DPAS, TS, CS, and CorS were determined in *N. benthamiana* leaves by expressing eGFP (N-terminal) and mRFP1 (C-terminal) constructs. Untagged eGFP and mRFP1 were used as markers. Leaf discs were excised and mounted in water 3-days post infiltration and imaged using a cLSM 880 (Zeiss, Oberkochen, Germany) equipped with a C-Apochromat 40x/1.2 water objective. An Argon laser was used to excite GFP at a wavelength of 488 nm with 1-10 % transmission and 700 PMT gain. A Helium-Neon laser was used to excite RFP at a wavelength of 543 nm with 10-20 % transmission and 700 PMT gain. Spectral detectors were set to 490-550 nm to detect GFP emissions and 550-650 nm for RFP emissions. Pixel dwell time was set to approximately 1 μ-second with 8-fold line averaging, and a Pinhole size of 1 Airy Unit. Transmitted light signal was measured in the GFP channel with a T-PMT gain of 500. Image contrast and brightness were edited in Image J[7].

Fluorescence Lifetime Imaging Microscopy – Forster Resonance Energy Transfer (FLIM-FRET)

Leaf discs from *N. benthamiana* leaves overexpressing eGFP and mRFP1 constructs were mounted in water 3-days post infiltration. FLIM imaging was performed using Time-Correlated Single-Photon Counting (TCSPC) as implemented on a Stellaris 8 FALCON STED (Leica) equipped with a HC PL APO 40x/1.25 glycerol immersion objective (Leica). A White Light Laser (WLL) (Leica) was used to excite GFP at a wavelength of 489 nm at 80MHz pulse frequency. GFP emission between 495 and 583 nm were recorded using a HyD-X detector until reaching a photon/pixel count of 1000. Additional channels were used to image RFP fluorescence and chlorophyl autofluorescence, using excitation at a wavelength of 590 and emission windows between 597-640 nm (HyD-S detector) for RFP and between 652-845 nm (HyD-X detector) for chlorophyl autofluorescence. Data was acquired and analyzed using Leica Application Suite X. GFP fluorescence lifetimes were calculated from FLIM images with a 100 photons/pixel threshold based on fluorescence decay model fitting using n-exponential reconvolution, as implemented in Leica Application Suite X. FRET efficiencies were calculated with equation 1 using amplitude weighted mean fluorescence lifetimes of GFP from six images representing three biological replicates.

1) $FRET\ efficiency = 1 - \tau_{quenched} / \tau_{unquenched}$

Goodness of fit was assessed from residual plots and only fits with Chi-squared values between 0.9-1.5 were accepted. A background FRET efficiency of <5% was observed and all such samples fit best to a bi-exponential decay model (n=2). Samples showing above background FRET efficiency (CHS + CHIL, and RFP-GFP fusion) fit best to a tri-exponential decay model (n=3), where the additional exponential component corresponds to the GFP population undergoing FRET.

Heterologous Expression and Protein Purification from *E. coli*

*Cr*DPAS, *Cr*TS (wild-type and mutants), and *Cr*CS (wild-type and mutants) constructs were cloned into pOPINF plasmid containing a N-terminal His_6 tag (as described above) and transformed into *E. coli* BL21 (DE3) (*ThermoFisher*) cells by heat shock at 42 °C for 30 seconds. Cells were plated on LB agar 100 µg/mL carbenicillin plates and grown overnight at 37 °C. A single colony was then picked and grown in 5 mL 2xYT media containing 100 µg/mL carbenicillin at 37 °C shaking at 200 r.p.m. overnight.

For protein purification, 1 mL of the overnight culture was added to 1L 2xYT media containing 100 µg/mL carbenicillin and grown at 37 °C shaking at 200 r.p.m. until OD_{600} = 0.6-0.8. Cultures were then transferred to an 18 °C incubator with shaking at 200 r.p.m for 30 minutes. Protein production was induced with 0.2 mM IPTG and incubated overnight for 18 hours. Cells were harvested by centrifugation at 3200 x g for 15 minutes and re-suspended in 50 mL buffer A1 (50 mM Tris-HCl pH 8, 50 mM glycine, 500 mM NaCl, 10 mM imidazole) and 50 mg lysozyme (*Sigma*) for 30 minutes at 37 °C. Cell suspensions were then chilled on ice for 15 minutes prior to lysis via Sonoplus HD2070 sonicator (50% amplitude, 1 second on, 1 second off, 3 min; *Bandeln*) and centrifuged (35,000 x g) to remove insoluble cell debris. The clarified lysate (supernatant) was collected, and the protein of interest purified using a gravity column containing 2 mL of Ni/NTA beads (*QIAGEN*). The clarified lysate was poured over the column twice and then the column was washed with 10 column volumes (CV) of buffer A1 before being eluted with 2 CV of buffer A1 + 250 mM imidazole. Proteins were buffer exchanged into 50 mM N-2-Hydroxyethylpiperazine-N-2-ethanesulfonic acid (HEPES) pH = 7.5 and concentrated using a 10 kDa molecular weight cut-off centrifugal filter (*Merck*) before being snap frozen in liquid nitrogen and stored at -70 °C.

In vitro Enzyme Assays

Enzymatic assays with precondylocarpine acetate were performed in 50 mM HEPES buffer (pH 7.5) with 1 mM precondylocarpine acetate in MeOH (not exceeding 5% of the reaction volume), 1 mM NADPH (*Sigma*), 0.2 μM of *Cr*DPAS, and 1.0 μM (*Cr*TS) or 3.0 μM (*Cr*CS) of competing cyclase enzymes to a final reaction volume of 50 μL. Substoichiometric ratios of *Cr*DPAS to cyclases were used to competition of the cyclases for dehydrosecodine.
Reactions were incubated for 20 minutes at 25 °C before being quenched 1:50 with MeOH. Reactions were filtered before being analyzed by UPLC-MS.
Enzymatic assays with angryline were performed in 50 mM N-cyclohexyl-2-aminoethanesulfonic acid (CHES) buffer (pH 9.5) with 1 mM angryline in MeOH (not exceeding 5% of the reaction volume) and 1 μM of each tested cyclase enzyme with a final reaction volume of 50 μL. Reactions were incubated for 10 minutes at 25 °C before being quenched 1:50 with MeOH. Reactions were filtered before being analyzed by UPLC-MS. *Reactions with CrCS variants were performed, but due to an impurity from the angryline substrate with the same m/z and near-identical retention time, analysis of the results was not fruitful. See Caputi et al. 2018* [2] *for more information.*

UPLC-MS

All assays were analyzed using a Thermo Scientific Vanquish UPLC coupled to a Thermo Q Exactive Plus orbitrap MS. Chromatographic separation was performed using a Phenomenex Kinetex C18 2.6 μm (2.1 x 100 mm) column using water with 0.1% formic acid as mobile phase A and acetonitrile with 0.1% formic acid as mobile phase B. Compounds were separated using a linear gradient of 10-30% B in 5 minutes followed by 1.5 minutes isocratic at 100% B. The column was then re-equilibrated at 10% B for 1.5 minutes. The column was heated to 40 °C and flow rate was set to 0.6 mL/min. MS detection was performed in positive ESI under the following conditions: spray voltage was set to 3.5 kV ~ 67.4 μA, capillary temperature set to 275 °C, vaporizer temperature 475 °C, sheath gas flow rate 65, sweep gas flow rate 3, aux gas flow rate 15, S-lens RF level to 55 V. Scan range was set to 200 - 1000 *m/z* and resolution at 17500.

Structural Modelling

The structural analyses carried out during this work were completed using the published crystal structures of *Cr*TS (PDB 6RS4), *Cr*CS (PDB 6RT8), and *Ti*CorS (PDB 6RJ8)[6], and Alphafold[8] structural predictions of *Cr*CorS. Structural predictions of *Cr*DPAS-*Cr*TS interactions were modelled using Alphafold3[9] with two copies of each respective enzyme, four zinc ions, and two copies of NADPH. Values for ipTM (0.48) and pTM (0.55) were below, or near to, the critical 0.6 (ipTM) and 0.5 (pTM) thresholds, indicating limited confidence in the predicted quaternary arrangements. Split-luciferase assays suggest a *Cr*DPAS-*Cr*TS interaction which is disrupted by the *Cr*TS-N219I variant. Importantly, the Alphafold3 predictions place N219 at the interface between *Cr*TS and *Cr*DPAS. Collectively, the low confidence Alphafold3 model and congruent biochemical characterization supports the existence of a transient DPAS-TS interface. We tentatively present the *Cr*DPAS-*Cr*TS model to visualize a *possible* interface which places DPAS and TS active site clefts in proximity.

Protein and DNA sequences

<u>CrCS</u>

Wildtype - DNA Sequence (NCBI: MF770512). Sites targeted for mutagenesis highlighted in red
atgaattcctcaactgatccaacttcagatgagactatttgggatctttctccatatattaaaattttcaaagatggaagagtagaaagactccata
atagtccttatgttcccccatcacttaatgatccagaaactggcgtttcttggaaagatgtcccgatttcatcacaagtttccgctagggtatacat
tccaaaaatcagcgaccatgaaaaactccctatttttgtgtatgtgcatggggctggcttttgtctagaatctgccttcagatcatttttccacactt
ttgtcaaacacttcgtagccgaaaccaaagttattggggtttcgattgaatatagacttgccccagagcaccttttacccgcagcttatgaagat
tgttgggaagcccttcaatgggttgcttctcatgtgggtctcgacaattccggcctaaagacagctattgataaagatccatggataataaacta
tggtgatttcgatagactgtatttggcgggtgacagtcctggtgctaatattgttcacaacacacttatcagagctggaaaagagaaactgaag
ggcggagtgaaaattttgggggcaattctttactacccatatttcattatcccaaccagcacgaaacttagtgatgattttgagtataactacaca
tgttactggaaattggcttatccaaatgctcctggcgggatgaataacccaatgataaaccccatagctgaaaatgctccagacttggctggat
acggttgctcgaggttgttggttaccctggtttccatgatttcaacgactccagatgagactaaagacataaatgcggtttatattgaggcattag
aaaagagtggatggaaaggggaattggaagtggctgattttgacgcagattattttgaactcttcaccttggaaacggagatgggcaagaat
atgttcagacgtttagcatctttcatcaaacatgagtaa

Y221I - DNA Sequence with mutated sites highlighted in red
atgaattcctcaactgatccaacttcagatgagactatttgggatctttctccatatattaaaattttcaaagatggaagagtagaaagactccata
atagtccttatgttcccccatcacttaatgatccagaaactggcgtttcttggaaagatgtcccgatttcatcacaagtttccgctagggtatacat
tccaaaaatcagcgaccatgaaaaactccctatttttgtgtatgtgcatggggctggcttttgtctagaatctgccttcagatcatttttccacactt
ttgtcaaacacttcgtagccgaaaccaaagttattggggtttcgattgaatatagacttgccccagagcaccttttacccgcagcttatgaagat
tgttgggaagcccttcaatgggttgcttctcatgtgggtctcgacaattccggcctaaagacagctattgataaagatccatggataataaacta
tggtgatttcgatagactgtatttggcgggtgacagtcctggtgctaatattgttcacaacacacttatcagagctggaaaagagaaactgaag
ggcggagtgaaaattttgggggcaattctttactacccatatttcattatcccaaccagcacgaaacttagtgatgattttgagataaactacaca
tgttactggaaattggcttatccaaatgctcctggcgggatgaataacccaatgataaaccccatagctgaaaatgctccagacttggctggat
acggttgctcgaggttgttggttaccctggtttccatgatttcaacgactccagatgagactaaagacataaatgcggtttatattgaggcattag

aaaagagtggatggaaaggggaattggaagtggctgattttgacgcagattattttgaactcttcaccttggaaacggagatgggcaagaat
atgttcagacgtttagcatctttcatcaaacatgagtaa

<u>CrTS</u>

Wildtype - DNA Sequence (NCBI: MF770513*). Sites targeted for mutagenesis highlighted in red*
atgggttcctcagatgagactatttttgatcttcctccatacatcaaagtcttcaaagatggaagagtagaaagactccattcttccccatatgttc
ccccatctcttaatgatccagaaaccggtggagtctcttggaaagacgtcccaatttcttcagtagtttcagctagaatttaccttcctaaaatca
acaaccatgatgaaaaactccccattatagtctatttccatggagctggtttttgtcttgaatcggccttcaaatcatttttccacacttatgtgaaa
cactttgtagcagaagccaaagctattgcggtttctgttgagttcaggctcgcccctgaaaaccatttacccgcagcttatgaagattgctggg
aagcccttcaatgggttgcttctcatgtgggtctcgacatttccagcttgaagacatgtattgataaagatccatggataatcaactatgccgatt
tcgatagactctatttgtggggtgatagcaccggtgccaatattgttcacaacacacttatcagatctggtaaagagaaattgaacggcggca
aagtgaagattttgggggcaattctttactacccatatttcttaatcaggacgagttcaaaacagagtgattatatggagaatgagtatagatctt
actggaaattggcttacccagatgctcctggtggaaatgataacccaatgataaaccctacagctgagaatgctcctgatctggctggatatg
gttgctcgaggttgctgatttccatggttgccgatgaagctagagatataactcttctttatattgatgcattggaaaagagtggatggaaaggt
gaattagatgtggctgattttgataaacagtattttgaactgtttgaaatggaaacagaggttgccaagaacatgctcagacgtttagcttctttca
tcaagtaa

N219I - DNA Sequence with mutated sites highlighted in red
atgggttcctcagatgagactatttttgatcttcctccatacatcaaagtcttcaaagatggaagagtagaaagactccattcttccccatatgttc
ccccatctcttaatgatccagaaaccggtggagtctcttggaaagacgtcccaatttcttcagtagtttcagctagaatttaccttcctaaaatca
acaaccatgatgaaaaactccccattatagtctatttccatggagctggtttttgtcttgaatcggccttcaaatcatttttccacacttatgtgaaa
cactttgtagcagaagccaaagctattgcggtttctgttgagttcaggctcgcccctgaaaaccatttacccgcagcttatgaagattgctggg
aagcccttcaatgggttgcttctcatgtgggtctcgacatttccagcttgaagacatgtattgataaagatccatggataatcaactatgccgatt
tcgatagactctatttgtggggtgatagcaccggtgccaatattgttcacaacacacttatcagatctggtaaagagaaattgaacggcggca
aagtgaagattttgggggcaattctttactacccatatttcttaatcaggacgagttcaaaacagagtgattatatggagatagagtatagatctt
actggaaattggcttacccagatgctcctggtggaaatgataacccaatgataaaccctacagctgagaatgctcctgatctggctggatatg
gttgctcgaggttgctgatttccatggttgccgatgaagctagagatataactcttctttatattgatgcattggaaaagagtggatggaaaggt
gaattagatgtggctgattttgataaacagtattttgaactgtttgaaatggaaacagaggttgccaagaacatgctcagacgtttagcttctttca
tcaagtaa

<u>CrCorS</u>

Wildtype - DNA Sequence (NCBI: PP133283*). Sites targeted for mutagenesis highlighted in red*
atggcttcccaaactccaacctcagatgagactcttttcgatctttctccatacatcagaatcttcaaagatggaagagtagaaagactccataa
tactccttatgttcccccatcacttaatgatccagaaaccggcgtctcttggaaagacgtcccaatttcatcaaaagtttcggctagaatttacctt
ccaaaaatcagtgaccagcaggaaaatgaagaaaaactcccaatttttgtttatttccatggggctggcttctgtctagaatctgcattcagatc
atttttccacacttttatcaaacactttgtatccgaagccaaagccattggggtttcggttgaatacagactcgccccggaacaccctttacccg
cagcttatgaagattgctgggaagcccttcaatgggtcgcttctcacgttcgtctcgacaattcaagcctcaagagatctatggacaaggatcc
atggataatcaactatggcgatttcgatagactctatttgggggggtgatagtcccggtggcaatattgttcacaacgtacttctcagagctggaa
aagagaaattgaatgggggagtgaaaattttgggggcaattcagtattacccatatttcctgatccggacgagctcgaaacagagtgattatat
ggagaatgactacaggtgttactggaaattggcttatccaaatgctcctggtggaactgataacccaatgataaaccccacagttgagaatgc
tcctgatttggccggatatggttgctccaggctgctgatttcaatggttgctgatgagactagagatataactctgctttttattgaggcattgaag
aagagcggatggaaagggcaattggatgtggctgattttgaagcagagtttttttgaccttttccaaacacaaacagaggtgggcaagaacat
gattagacgcttaacgtctttcatcaaatga

M^4 (N32D, S216K, N224I, E301H) - DNA Sequence with mutated sites highlighted in red
atggcttcccaaactccaacctcagatgagactcttttcgatctttctccatacatcagaatcttcaaagatggaagagtagaaagactccatga
tactccttatgttcccccatcacttaatgatccagaaaccggcgtctcttggaaagacgtcccaatttcatcaaaagtttcggctagaatttacctt
ccaaaaatcagtgaccagcaggaaaatgaagaaaaactcccaatttttgtttatttccatggggctggcttctgtctagaatctgcattcagatc
atttttccacacttttatcaaacactttgtatccgaagccaaagccattggggtttcggttgaatacagactcgccccggaacacccctttacccg
cagcttatgaagattgctgggaagcccttcaatgggtcgcttctcacgttcgtctcgacaattcaagcctcaagagatctatggacaaggatcc
atggataatcaactatggcgatttcgatagactctatttgggggggtgatagtcccggtggcaatattgttcacaacgtacttctcagagctggaa
aagagaaattgaatgggggagtgaaaattttgggggcaattcagtattacccatatttcctgatccggacgagcaaaaaacagagtgattata
tggagatagactacaggtgttactggaaattggcttatccaaatgctcctggtggaactgataacccaatgataaaccccacagttgagaatg
ctcctgatttggccggatatggttgctccaggctgctgatttcaatggttgctgatgagactagagatataactctgctttttattgaggcattgaa
gaagagcggatggaaagggcaattggatgtggctgattttgaagcacactttttgaccttttccaaacacaaacagaggtgggcaagaaca
tgattagacgcttaacgtctttcatcaaatga

M^5 (D24N, R26K, E72K, F167L, I281L) - DNA Sequence with mutated sites highlighted in red
atggcttcccaaactccaacctcagatgagactcttttcgatctttctccatacatcagaatcttcaaaaacggaaaggtagaaagactccataa
tactccttatgttcccccatcacttaatgatccagaaaccggcgtctcttggaaagacgtcccaatttcatcaaaagtttcggctagaatttacctt
ccaaaaatcagtgaccagcagaagaatgaagaaaaactcccaatttttgtttatttccatggggctggcttctgtctagaatctgcattcagatc
atttttccacacttttatcaaacactttgtatccgaagccaaagccattggggtttcggttgaatacagactcgccccggaacacccctttacccg
cagcttatgaagattgctgggaagcccttcaatgggtcgcttctcacgttcgtctcgacaattcaagcctcaagagatctatggacaaggatcc
atggataatcaactatggcgatctcgatagactctatttgggggggtgatagtcccggtggcaatattgttcacaacgtacttctcagagctgga
aaagagaaattgaatgggggagtgaaaattttgggggcaattcagtattacccatatttcctgatccggacgagctcgaaacagagtgattat
atggagaatgactacaggtgttactggaaattggcttatccaaatgctcctggtggaactgataacccaatgataaaccccacagttgagaat
gctcctgatttggccggatatggttgctccaggctgctgatttcaatggttgctgatgagactagagatataactctgctttttcttgaggcattga
agaagagcggatggaaagggcaattggatgtggctgattttgaagcagagtttttttgaccttttccaaacacaaacagaggtgggcaagaac
atgattagacgcttaacgtctttcatcaaatga

CrDPAS
Wildtype - DNA Sequence (NCBI: KU865331*)*
atggccggaaaatcagcagaagaagaacatcccattaaggcttacggatgggctgttaaagatagaacaactgggattctttctcccttcaaa
ttttccagaagggcaacaggtgatgatgatgtccgaattaagatactctactgtggaatttgtcacactgatcttgcctcaatcaagaacgaata
cgagtttctttcttatcctcttgtgcccgggatggagatcgttggaatagcaacggaggttggaaaagatgtcacaaaagtgaaagttggcga
aaaagtagcattatcagcctatttaggatgttgtggcaaatgctatagttgtgtaaatgaactcgagaattattgtccggaagtaatcataggttat
ggcaccccataccatgacggaacaatttgctatgggggcctttcaaacgaaactgtcgcaaatcaaagttttgttcttcgttttcctgaaagact
ttctccagctggcggagctcctttgcttagcgccggaattacttcgtttagtgcaatgagaaatagcggcatcgacaaacctggattacacgtg
ggagtcgtcggtctcggcggattaggtcatcttgctgtaaaatttgctaaggcttttggtcttaaagtaactgttattagcaccactcccagcaag
aaggatgatgctataaatggtcttggtgctgatggattcttactcagccgcgatgatgaacaaatgaaggctgctattggaaccttggatgcaa
ttattgatacactggcggttgttcatcccatagcaccattgcttgatctcctgagaagtcaagggaaatttttgttacttggggcgccatctcaatc
acttgagttgccacctattcctttattatcaggtgggaaatctatcattggaagtgcggccggaaatgtgaagcaaactcaagaaatgcttgatt
ttgcagcggagcatgatataactgcaaatgttgagattattccaatagagtacataaatactgcaatggaacgtttagacaagggcgatgttag
ataccgatttgtagttgacatcgaaaataccttgactcctccgtcagagttataa

TiCorS

Wildtype - DNA Sequence (NCBI: MK840854*). Sites targeted for mutagenesis highlighted in red*

atggctaattcaactgcaaactctgatgagattgttttcgatcttcatccatacatcagagtctttaaaaacggcaaggtagaaagacttcacga
caccccatatgttccgccatcacttgaagatccagccaccggtgtatcctggaaagacgtcccaatttcatccgacgtttcagctagagtctac
ctcccgaagatcagcgaagcggaaaagaaaaagctccccattttcgtctatttccatggtgcaggcttctgtctggaatcagccttcaaatcat
tttccatacttatgttaagcacgttgttgccgaaaccaaagctgtcggagtttcggttgagtacagactcgcccccgagcaccctttacctgcg
gcttatgaagattgctggactgcccttcagtgggtggcttcccatgttggtcttgacaactccagcctcaagaatgctattgataaagagccttg
gataatcaaccatggcgacctcaataagctttacttgggtggtgacagtcctggtggaaatattgtgcacaacgtactgattagagctggtaag
gagagcttgcatggcggagtgaaaatccggggtgcaattctttattacccatatttcttgatcaggacaagcaaaagacagagtgattatatgg
agattgactatagaggctactggaagttggcttatccatctgctcctggcggcactgacaacccaatgataaaccctgtagctaagaatgctc
ctgatttggccggatatggatgttcgaggctgcttgtttccatggtttcggacgagaccagagatataacccttctctaccttgaggcattgaag
aagagtgggtggaaaggtgaattggaagtgggtgactacgaagcacatttctttgatttgttcagccctgaaaatgaagttggcaagacttgg
atcaaacgttcaagcgatttcatcaacaaggagtag

M^4 (D32N, K214S, I222N, H299E) - DNA Sequence with mutated sites highlighted in red

atggctaattcaactgcaaactctgatgagattgttttcgatcttcatccatacatcagagtctttaaaaacggcaaggtagaaagacttcacaat
accccatatgttccgccatcacttgaagatccagccaccggtgtatcctggaaagacgtcccaatttcatccgacgtttcagctagagtctacc
tcccgaagatcagcgaagcggaaaagaaaaagctccccattttcgtctatttccatggtgcaggcttctgtctggaatcagccttcaaatcattt
ttccatacttatgttaagcacgttgttgccgaaaccaaagctgtcggagtttcggttgagtacagactcgcccccgagcaccctttacctgcgg
cttatgaagattgctggactgcccttcagtgggtggcttcccatgttggtcttgacaactccagcctcaagaatgctattgataaagagccttgg
ataatcaaccatggcgacctcaataagctttacttgggtggtgacagtcctggtggaaatattgtgcacaacgtactgattagagctggtaagg
agagcttgcatggcggagtgaaaatccggggtgcaattctttattacccatatttcttgatcaggacaagctccagacagagtgattatatgga
gaatgactatagaggctactggaagttggcttatccatctgctcctggcggcactgacaacccaatgataaaccctgtagctaagaatgctcc
tgatttggccggatatggatgttcgaggctgcttgtttccatggtttcggacgagaccagagatataacccttctctaccttgaggcattgaaga
agagtgggtggaaaggtgaattggaagtgggtgactacgaagcagaattctttgatttgttcagccctgaaaatgaagttggcaagacttgg
atcaaacgttcaagcgatttcatcaacaaggagtag

M^5 (N24D, K26R, K72E, L165F, Y278F) - DNA Sequence with mutated sites highlighted in red

atggctaattcaactgcaaactctgatgagattgttttcgatcttcatccatacatcagagtctttaaagatggcagagtagaaagacttcacgac
accccatatgttccgccatcacttgaagatccagccaccggtgtatcctggaaagacgtcccaatttcatccgacgtttcagctagagtctacc
tcccgaagatcagcgaagcggaagaaaaaaagctccccattttcgtctatttccatggtgcaggcttctgtctggaatcagccttcaaatcattt
ttccatacttatgttaagcacgttgttgccgaaaccaaagctgtcggagtttcggttgagtacagactcgcccccgagcaccctttacctgcgg
cttatgaagattgctggactgcccttcagtgggtggcttcccatgttggtcttgacaactccagcctcaagaatgctattgataaagagccttgg
ataatcaaccatggcgacttcaataagctttacttgggtggtgacagtcctggtggaaatattgtgcacaacgtactgattagagctggtaagg
agagcttgcatggcggagtgaaaatccggggtgcaattctttattacccatatttcttgatcaggacaagcaaaagacagagtgattatatgga
gattgactatagaggctactggaagttggcttatccatctgctcctggcggcactgacaacccaatgataaaccctgtagctaagaatgctcct
gatttggccggatatggatgttcgaggctgcttgtttccatggtttcggacgagaccagagatataacccttctctacattgaggcattgaagaa
gagtgggtggaaaggtgaattggaagtgggtgactacgaagcacatttctttgatttgttcagccctgaaaatgaagttggcaagacttggat
caaacgttcaagcgatttcatcaacaaggagtag

TiTabS

Wildtype - DNA Sequence (NCBI: MK840853*). Sites targeted for mutagenesis highlighted in red*

atggcttcttcaactgaaagctctgatgagattatttttgatcttcctccatacattagagtctttaaggatggaagagtagagagactccactcct
caccatatgttccaccatcactagatgatcccgcaaccggcgtatcctggaaagacgtcccaatttcatcagaggtttcggctagaatctacct
cccaaagataagccaaaaggaaaaggaaaagcttcccattgtggtctatttccatggtgcaggcttctgtctggaatccgcctacaagtcattt
ttccacacttatgtcaagcactttgcagccgaggccaaagcaattgcagtttcggttgagttcaggctctccccagagcaccacctgcctgca
gcttatgaagattgctggactgcccttcagtgggtggcttcacatgtagatgttgacaactccagcctcaagaatgctatagataaagagcctt
ggataatcaaccatggagactttgacaagatctacttatggggtgacagtacgggtgccaatattgtgcacaacgtactcatcagagctggta
atgagagcttgcatggcggagtgaaaatcgtgggtgcaattctttattacccatatttcttgatcaggacaagctccagacagagcgattatat
ggagaacgagtacagagcatactggaagctggcttatccatctgctccaggtgggaacgacaacccgatgataaaccccgtagctgagaa
cgctcctgatttggctggatatggatgttcgaggctgctggtatccatggtggcagacgaggccagagacataacccttctctacatcgagg
cagtgaagaagagtgggtggaaaggtgaattggaggtggctgatttcgaaggagattactttgaaatattcagcccagaaactgagacagg
caagaacaaggtcaaacgtttaacgtctttcatcaacaaggagtag

N224I - DNA Sequence with mutated sites highlighted in red

atggcttcttcaactgaaagctctgatgagattatttttgatcttcctccatacattagagtctttaaggatggaagagtagagagactccactcct
caccatatgttccaccatcactagatgatcccgcaaccggcgtatcctggaaagacgtcccaatttcatcagaggtttcggctagaatctacct
cccaaagataagccaaaaggaaaaggaaaagcttcccattgtggtctatttccatggtgcaggcttctgtctggaatccgcctacaagtcattt
ttccacacttatgtcaagcactttgcagccgaggccaaagcaattgcagtttcggttgagttcaggctctccccagagcaccacctgcctgca
gcttatgaagattgctggactgcccttcagtgggtggcttcacatgtagatgttgacaactccagcctcaagaatgctatagataaagagcctt
ggataatcaaccatggagactttgacaagatctacttatggggtgacagtacgggtgccaatattgtgcacaacgtactcatcagagctggta
atgagagcttgcatggcggagtgaaaatcgtgggtgcaattctttattacccatatttcttgatcaggacaagctccagacagagcgattatat
ggagatagagtacagagcatactggaagctggcttatccatctgctccaggtgggaacgacaacccgatgataaaccccgtagctgagaa
cgctcctgatttggctggatatggatgttcgaggctgctggtatccatggtggcagacgaggccagagacataacccttctctacatcgagg
cagtgaagaagagtgggtggaaaggtgaattggaggtggctgatttcgaaggagattactttgaaatattcagcccagaaactgagacagg
caagaacaaggtcaaacgtttaacgtctttcatcaacaaggagtag

TiDPAS1

Wildtype - DNA Sequence (NCBI: MK840855*)*

atggctgtaaaatcacctgaagcagagcacccagtgaaggctgtaaaatcacctgaagaagagcacccagtgaaggcatacggatgggc
tatcaaagacagaacatctggcattctttccccccttcaagttttccagaagggcaacaggagatgaagacgttcgaataaagatcctctgttgc
ggagtttgtcacacggatctcacgtctaccaagaatgaatacgagtttctttcatatcctctagtgcctgggttggagactgtcggaatagcga
cagaggtcggaagcaaagtcacaaaagtaaaagttggtgaaaaagtagcagtggcagcctatttgggcacttgtggcaaatgccacaattg
tctaaatgaccaagagaattactgtcccgaagtgatcattagctacggcacaccatatcacgacggaacaatcaactacggaggcttctcga
atgagacggtcgtaaatgagcgcttcgttcttcattttcctgaaaagctttcactttctggtggtgcaccgctactcagcgcaggaagcaccgct
tacagtgcaataagaaatcaaggccttgacaaacccggtatccacttgggagtcgtcggccttggtggacttggtcatctggctgtgaagttt
gccaaggcttttggtgtcaaggtgacagtgattagttccactcccagcaagaaggatgaagccatcaagagccttggtgccgatgcgttcttg
ttcagtcgtgatgatgaacaaatgaaggccgctattggaactttcgatgcaatcatagatactattgcagtcgctcatcctcttgcgccattactt
gatctactaaggagtcatggaaaaattattttggtcggggcaccgaccaccccacttgaggtgccagttattcctttagtagcaggtgggaaat
cgattactggatgcgtagttggaaatttgaagcaaactcaagaaatgcttgaatttgctgcagaacacaacatcactgcaaacgttgaggttat
ttcaatggattacataaacactgcaatggaacgtttagaaaaaggtgatgttagatatagatttgtaattgatattggaaacacactaactccacc
ggaatag

<u>TiDPAS2</u>

Wildtype - DNA Sequence (NCBI: MK840856*)*

atggcaggaaaatcaccagaagaggaacacccagtgaaggcatatggatgggctgtcaaggacagaacaactgggattctttctcccttca
agttttcgagaagggcaacaggagacaatgacatccgaatcaagatcctctattgtggaatttgtcatacagacctaacatctgtcaagaacg
agtacgagtttctttcatatcctcttgtgcctgggatggagattgtagggatagcgacagaggtcggaagcaaagtcacaaaaataaaagttg
gtgaaaaagtagcagtagcagcctatttgggtacttgtggaaaatgctacaattgtgtaaatgaccttgagaactactgtcctgaagtcatcatt
ggttatggtacgccatatcacgatggaacaattaactacggaggcctctcaaacgagacggtcgtaaatgagcgctttgttcttcgttttcctga
aaaactttcacctgctggtggtgccccgctactcagtgctggaatcaccgcgtacagtgcaatgaggaatcatggcctcgacaagcccgga
atccaattgggagtcgtcggtcttggtggacttggtcatctggctgtgaagtttgccaaggcttttggcgtcagagtgactgtgattagtaccac
tcctagcaagaaggatgaagctataaataatcttggtgctgatgcctttttgttcagccgtgacgataagcaaatgagggctgccattggaacg
tttgatgcaatcatcgacacactagcggttgttcatcctattgcgcccttactcgatctattgaggagtcatggaaaacttgttttggttggagccc
catctaagccacttgagctaccaactattcctttattatcaggaggaaaatcattgatcggtagtgcagctggaaatgtgaagcaaactcagga
aatgcttgattttgcagcagaacacgatattactgcaaacattgaggttataccaatagattatataaacactgcaatggaacgtttagataaag
gtgatatccgatttaggtttgtggttgatattgaaaataccttaactcctccgccagaaccgtaa

Supplementary References

1. Ban, Z. *et al.* Noncatalytic chalcone isomerase-fold proteins in Humulus lupulus are auxiliary components in prenylated flavonoid biosynthesis. *Proc. Natl. Acad. Sci. U. S. A.* **115**, E5223–E5232 (2018).
2. Caputi, L. *et al.* Missing enzymes in the biosynthesis of the anticancer drug vinblastine in Madagascar periwinkle. *Science* **360**, 1235–1239 (2018).
3. Kamileen, M. O. *et al.* Recycling Upstream Redox Enzymes Expands the Regioselectivity of Cycloaddition in Pseudo-Aspidosperma Alkaloid Biosynthesis. *J. Am. Chem. Soc.* (2022) doi:10.1021/jacs.2c08107.
4. Li, C. *et al.* Single-cell multi-omics in the medicinal plant Catharanthus roseus. *Nat. Chem. Biol.* **19**, 1031–1041 (2023).
5. Liscombe, D. K. & O'Connor, S. E. A virus-induced gene silencing approach to understanding alkaloid metabolism in Catharanthus roseus. *Phytochemistry* **72**, 1969–1977 (2011).
6. Caputi, L. *et al.* Structural basis of cycloaddition in biosynthesis of iboga and aspidosperma alkaloids. *Nat. Chem. Biol.* **16**, 383–386 (2020).
7. Schindelin, J. *et al.* Fiji: an open-source platform for biological-image analysis. *Nat. Methods* **9**, 676–682 (2012).
8. Jumper, J. *et al.* Highly accurate protein structure prediction with AlphaFold. *Nature* **596**, 583–589 (2021).
9. Abramson, J. *et al.* Accurate structure prediction of biomolecular interactions with AlphaFold 3. *Nature* **630**, 493–500 (2024).
